# Trends in Overweight and Obesity among Children and Adolescents in China from 1981 to 2010: A Meta-Analysis

**DOI:** 10.1371/journal.pone.0051949

**Published:** 2012-12-17

**Authors:** Zhangbin Yu, Shuping Han, Jiahui Chu, Zhongya Xu, Chun Zhu, Xirong Guo

**Affiliations:** 1 State Key Laboratory of Reproductive Medicine, Department of Pediatrics, Nanjing Maternity and Child Health Care Hospital, Nanjing Medical University, Nanjing, China; 2 The First School of Clinical Medicine, Nanjing Medical University, Nanjing, China; University of Montreal, Canada

## Abstract

**Background:**

Overweight/obesity is a serious public health problem that affects a large part of the world population across all age and racial/ethnic groups. However, there has not been a meta-analysis of the prevalence of childhood and adolescent overweight/obesity in China during the past 30 years.

**Methods:**

The China National Knowledge Infrastructure and Wanfang DATA, MEDLINE, EMBASE and Cumulative Index to Nursing and Allied Health Literature were searched for relevant studies published between January 1970 and June 2012. The prevalence of overweight/obesity over time was pooled using Stata/SE, version 9. Summary statistics (odds ratios, ORs) were used to compare sex-specific and urban-rural preponderance of overweight/obesity using Review Manager.

**Results:**

After screening 1326 papers, we included 35 papers (41 studies), most of medium quality. The prevalence of overweight/obesity increased from 1.8% (95% confidence interval [CI], 0.4%–3.1%) and 0.4% (95% CI, −0.1% to −0.8%) respectively in 1981–1985 to 13.1% (95% CI, 11.2%–15.0%) and 7.5% (95% CI, 6.6%–8.4%) respectively in 2006–2010. The average annual increase was 8.3% and 12.4% respectively. Boys were more likely to be overweight/obese than girls (OR, 1.36; 95% CI, 1.24–1.49 and OR, 1.68; 95% CI, 1.52–1.86 respectively). The prevalence of overweight/obesity was higher in urban areas than in rural areas (OR, 1.66; 95% CI, 1.54–1.79 and OR, 1.97; 95% CI, 1.68–2.30 respectively). For age-specific subgroup analyses, both overweight and obesity increased more rapidly in the toddler stage than in other developmental stages. Sensitivity analyses showed that sample-size differences, study quality, overweight/obesity criteria and geographical distribution affected overweight/obesity prevalence.

**Conclusions:**

Toddlers and urban boys were at particularly high risk; the prevalence in these groups increased more rapidly than in their counterparts. Public health prevention strategies are urgently needed to modify health behaviors of children and adolescents and control overweight/obesity in China.

## Introduction

Overweight/obesity is a serious public health problem that affects a large part of the world population across all age, gender and racial/ethnic groups [1]. Overweight/obesity in the pediatric population has attracted much attention because childhood and adolescence are critical developmental periods during which individuals establish the foundations for their future health. Moreover, overweight/obesity in this age group could carry over to adulthood [2]. Childhood and adolescence is a brief, yet critical, window of opportunity for the prevention of the overweight/obesity epidemic.

Overweight/obesity in children and adolescents can cause developmental problems, such as poor cognitive function [3], psychological disorders [4], and altered timing of puberty [5], and may be accompanied by cardiovascular risk factors and metabolic syndrome [6]. Overweight/obesity during childhood and adolescence is also a risk factor for adult insulin resistance [7], diabetes mellitus [8], hypertension [9], coronary heart disease and stroke [10], [11], and even mortality [12].

With ongoing, rapid urbanization and industrialization, many developing countries are facing a significant and rapidly growing epidemic of overweight/obesity in children and adolescents [13]. In China, recent, rapid economic growth has been associated with an increasing incidence of childhood and adolescent overweight/obesity [14]. A growing interest in monitoring overweight/obesity has led to a number of studies, including a few national surveys, which have investigated the prevalence of these conditions in Chinese children and adolescents [15]. These studies provide updated health information for the development of effective programs and strategies to prevent and control overweight/obesity. However, there has been no summary or critical appraisal of the body of literature on the prevalence of childhood and adolescent overweight/obesity in China published during the past 30 years. Prior reviews on this topic have been limited by a focus on a certain national survey [16], [17]. It is difficult to predict trends in overweight and obesity in Chinese children and adolescents because of the insufficient and non-representative samples in national surveys, the findings of which may not be applicable to the entire population. Therefore, we extended this review to a larger number of studies and national surveys, carried out a meta-analysis of the prevalence of overweight and obesity from infancy to adolescence in China and described the secular trends in this prevalence from 1981 to 2010 and the trends in the prevalence categorized by place of residence (urban-rural) and gender.

## Methods

This systematic review and meta-analysis was conducted according to the guidelines for the Meta-analysis of Observational Studies in Epidemiology [18] and the Preferred Reporting Items for Systematic Reviews and Meta-analyses (PRISMA) statement.

### Study selection criteria

Studies were required to conform to the following criteria: (i) a sample that included children and adolescents (ages, 0–18 years), (ii) cohort and cross-sectional design, (iii) original studies that presented the prevalence of overweight/obesity and (iv) study setting in China. Studies that defined overweight/obesity categories according to body mass index (BMI) were included. BMI was calculated by dividing body weight (kg) by the square of the height (m^2^). The Centers for Disease Control and Prevention (CDC, USA) [19], International Obesity Task Force (IOTF) [20] and Working Group for Obesity in China (WGOC) [21] have separately published BMI reference standards for children and adolescents, and overweight was defined as BMI >85^th^ percentile but ≤95^th^ percentile, related to gender and age, whereas obesity was defined as BMI >95^th^ percentile. Studies that classified overweight/obesity according to the deviation from the ideal weight-for-height recommended by the World Health Organization (WHO) were also included. In these studies, the ratio of weight (W) to ideal weight (IW) was calculated; overweight was defined as W/IW >1.1, and obesity, as W/IW >1.2 [22].

### Data sources and search strategies

Five electronic bibliographic databases, the Chinese journal full-text database of the China National Knowledge Infrastructure (CNKI), and the databases of Wanfang DATA, MEDLINE, EMBASE and Cumulative Index to Nursing and Allied Health Literature (CINAHL), were searched systematically for studies published between January 1970 and June 2012. The following terms, adapted for each database, were used for the searches: ‘incidence’ OR ‘frequency’ OR ‘prevalence’ OR ‘epidemiology’ AND ‘obesity’ OR ‘overweight’ OR ‘body mass index’ OR ‘BMI’ OR ‘weight gain’ AND ‘China’ OR ‘Chinese’ AND ‘infant’, ‘childhood’, ‘children’, ‘toddler’, ‘adolescence’, ‘adolescents’, ‘youth’, ‘teen’ and ‘teenager’ (Appendix S1). No restrictions on language were applied. Searching of gray literature and hand searching were not performed. If the data in the original publication lacked sufficient detail, the study authors were contacted for additional information.

### Screening and data-extraction form

Titles and abstracts were examined for inclusion by two independent reviewers (ZB Yu and JH Chu) on the basis of pre-defined inclusion criteria, and disagreements were resolved by consensus or referred to a third reviewer (SP Han), if necessary. For articles with relevant citations or with titles/abstracts with insufficient information for deciding on inclusion/exclusion, the full text was retrieved and evaluated. The following study characteristics were extracted from the articles: publication year, study time period, study design, representativeness of target population, sample selection, sample size, response rate, reasons for nonresponse, data source, data collection, description of obesity/overweight/sex/age/urban and rural, prevalence recall periods, study objectives, criteria for obesity or overweight and raw figures that allowed calculation of obesity/overweight prevalence. An independent reviewer (C Zhu) confirmed all data entries. Missing raw data were requested from authors by email.

### Assessment of study quality

To assess the quality of the included studies, we created a specific checklist (Appendix S2) based on the methodological criteria proposed by Leboeuf-Yde and Lauritsen for the assessment of prevalence studies, with relevant revisions [23]. In brief, we assessed the quality of all included studies on the basis of the following: study design, representativeness of target population, sample selection, sample size, response rate, reasons for nonresponse, data source and study objectives, data collection, description of obesity/overweight/sex/age/urban and rural and prevalence recall periods. Study quality was scored on a scale of 0 to 19, and studies were classified as high (>14), medium (11–14) or low (<11) quality [23].

### Statistical analysis

To permit comparison, studies that categorized data by residence (urban and rural) as well as by sex were tabulated separately. Studies were also tabulated by year of fieldwork. Because of insufficient numbers of studies for individual years, studies were grouped into six 5-year periods, 1981–1985, 1986–1990, 1991–1995, 1996–2000, 2001–2005 and 2006–2010. Date of fieldwork is a more reliable indicator of time trends than date of publication, which is variably delayed. Therefore, studies for which dates of fieldwork could not be ascertained were excluded from the time trend analysis. We calculated the prevalence of overweight/obesity over time, sex and residence (urban or rural) in terms of proportions and 95% confidence interval (CI) for each study, and pooled the data to derive a pooled proportion and 95% CI using Stata/SE, version 9 (Stata Corp., College Station, Texas, USA). Summary statistics (odds ratios, ORs) were used to compare sex-specific and urban-rural preponderance of overweight/obesity using Review Manager, version 5.1.7 (Nordic Cochrane Center, Copenhagen, Denmark). The chi-square test was used to analyze heterogeneity across studies. A random-effects model was used to account for possible heterogeneity between studies; a fixed-effects model was used in the absence of heterogeneity [24]. *P*-values<0.05 were considered significant. First, we assessed the prevalence of overweight/obesity in children and adolescents aged 0–18 years. Second, subgroup analyses were performed for the following growth and developmental stages: infancy, age<1 year; toddler, 1–3 years; pre-school, 4–6 years; school, 7–13 years; and adolescence, 14–18 years. Sensitivity analysis was performed to determine whether differences in sample size, study quality, diagnostic criteria for obesity/overweight and geographical distribution affected study conclusions. Publication bias was assessed by inspection of funnel plots, and formal testing for funnel plot asymmetry was performed using the Begg test and Egger test [25], which was carried out using Stata/SE.

## Results

### Literature search and study quality

**Figure 1:**
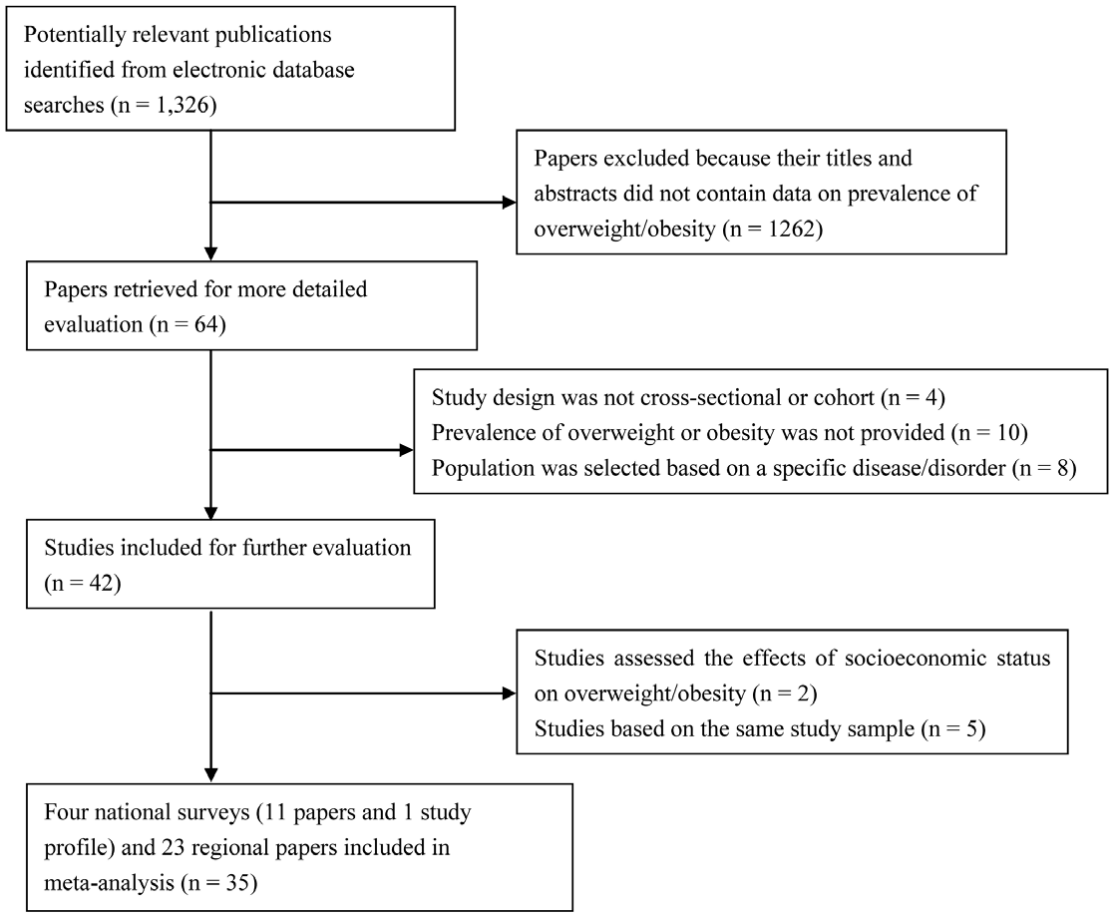
Flow chart of article screening and selection process.

A search of five electronic databases identified 1,326 papers, 1291 of which were excluded owing to the reasons listed in Figure 1. Thus, 35 papers (41 studies) were included in the meta-analysis [26]–[60], including four national surveys (12 papers) [26]–[37] and 23 regional papers [38]–[40]. Among the included papers, one national survey (China Health and Nutrition Survey, CHNS) has been conducted successively in 1989, 1991, 1993, 1997, 2000, 2004, 2006, their data can be obtained from the website http://www.cpc.unc.edu/projects/china/data/data.html. One paper [29] described the successive national survey included seven studies of CHNS in 1989, 1991, 1993, 1997, 2000, 2004, 2006. The other 34 papers described the corresponding 34 studies. Thus, 35 papers correspond with 41 studies in the meta-analysis. The prevalence of overweight/obesity, general information and study designs of the four national surveys, National Survey on Childhood Obesity (NSCO), Chinese National Survey on Students Constitution and Health (CNSSCH), Chinese National Nutrition and Health Survey (CNNHS) and CHNS, have been described in greater detail in Appendix S3. Three regional studies (Beijing City, Urumqi City and Shandong Province) from the sixth CNSSCH in 2010 were included [38]–[40]. In addition, 20 papers (20 studies) were included from regional surveys on overweight/obesity in children and adolescents [41]–[60]. Descriptive information for each study is presented in Table S1.

The quality of each paper based on 10 items is summarized in Appendix S4. NSCO and CNSSCH received scores of 15 and 16, respectively, and were considered to be of high methodological quality. CHNS and CNNHS received scores of 12 and 14, respectively, and were considered to be of medium methodological quality. Three regional studies from the sixth CNSSCH in 2010 received scores of 11–14, and were considered to be of medium methodological quality. Of the 20 papers from regional surveys on childhood overweight/obesity, six received scores of 11–14 and were considered to be of medium methodological quality; 14 papers received scores of ≤10 and were considered to be of low methodological quality. The PRISMA statement see Checklist S1.

### Meta-analysis of overweight/obesity prevalence in Chinese children and adolescents

Thirty-seven studies assessed the prevalence of overweight in a total of 2,016,361 Chinese children and adolescents (Table 1). A pooled analysis based on years of fieldwork categorized in five-year periods demonstrated an increase in overweight over time (Table 1). The prevalence of overweight increased from 1.8% (95% CI, 0.4%–3.1%) in all subjects, 1.6% (95% CI, 0.4%–2.7%) in boys and 2.0% (95% CI, 0.5%–3.6%) in girls in 1981–1985 to 13.1% (95% CI, 11.2%–15.0%) in all subjects, 15.4% (95% CI, 13.3%–17.5%) in boys and 10.7% (95% CI, 8.8%–12.6%) in girls in 2006–2010. These values represent 7.3-fold, 9.6-fold and 5.4-fold increases, respectively since 1981–1985, with average annual increase rates of 8.3%, 9.5% and 6.9%, respectively.

**Table 1:** Summary of studies reporting the prevalence of overweight in Chinese children and adolescents aged 0–18 years.

Author, year	Time period	Sample size (n)			Overweight (n)			Overweight, Prevalence, % (95% CI)		
	(years)	Boys	Girls	Total	Boys	Girls	Total	Boys	Girls	Total
**1981–1985**										
Chen.1986 (30)	1982	5334	4793	10127	51	60	111	1.0% (0.7%, 1.2%)	1.3% (0.9%, 1.6%)	1.1% (0.9%, 1.3%)
CNSSCH 1987 (33)	1985	205100	204846	409946	4406	5743	10149	2.1% (2.1%, 2.2%)	2.8% (2.7%, 2.9%)	2.5% (2.4%, 2.5%)
**Sub-total**		**210434**	**209639**	**420073**	**4457**	**5803**	**10260**	**1.6% (0.4%, 2.7%)**	**2.0% (0.5%, 3.6%)**	**1.8% (0.4%, 3.1%)**
**1991–1995**										
CHNS 1991(29)	1991	1333	1248	2581	47	50	97	3.5% (2.5%, 4.5%)	4.0% (2.9%, 5.1%)	3.8% (3.0%, 4.5%)
CNSSCH 1993 (34)	1991	70608	70047	140655	2798	2528	5326	4.0% (3.8%, 4.1%)	3.6% (3.5%, 3.7%)	3.8% (3.7%, 3.9%)
Ge.1995 (31)	1992	13251	11987	25238	477	366	843	3.6% (3.3%, 3.9%)	3.1% (2.7%, 3.4%)	3.3% (3.1%, 3.6%)
CHNS 1993 (29)	1993	1248	1144	2392	64	21	85	5.1% (3.9%, 6.4%)	1.8% (1.1%, 2.6%)	3.6% (2.8%, 4.2%)
CNSSCH 1997 (35)	1995	103960	104676	208636	5078	5317	10395	4.9% (4.8%, 5.0%)	5.1% (4.9%, 5.2%)	5.0% (4.9%, 5.1%)
**Sub-total**		**190400**	**189102**	**379502**	**8464**	**8282**	**16746**	**4.2% (3.6%, 4.8%)**	**3.5% (2.6%, 4.5%)**	**3.9% (3.1%, 4.7%)**
**1996–2000**										
Ding *et al.* 1998 (27)	1996	110993	97520	208513	4651	4019	8670	4.2% (4.1%, 4.3%)	4.1% (4.0%, 4.2%)	4.2% (4.1%, 4.3%)
CHNS 1997 (29)	1997	1269	1120	2389	67	56	123	5.3% (4.0%, 6.5%)	5.0% (3.7%, 6.3%)	5.1% (4.3%, 6.0%)
Hui *et al.* 2003 (43)	1999	2428	2146	4574	359	208	567	14.8% (13.4%, 16.2%)	9.7% (8.4%, 10.9%)	12.4% (11.4%, 13.4%)
CHNS 2000 (29)	2000	1216	1074	2290	86	51	137	7.1% (5.6%, 8.5%)	4.7% (3.5%, 6.0%)	6.0% (5.0%, 7.0%)
CNSSCH 2002 (36)	2000	111853	111919	223772	10536	8087	18623	9.4% (9.2%, 9.6%)	7.2% (7.1%, 7.4%)	8.3% (8.2%, 8.4%)
Chen *et al.* 2002 (44)	2000	1746	1611	3357	170	159	329	9.7% (8.3%, 11.1%)	9.9% (8.4%, 11.3%)	9.8% (8.8%, 10.8%)
**Sub-total**		**229505**	**215390**	**444895**	**15869**	**12580**	**28449**	**8.4% (5.4%, 11.4%)**	**6.7% (4.9%, 8.6%)**	**7.6% (5.2%, 10.0%)**
**2001–2005**										
Li *et al.* 2007 (45)	2001	2848	2840	5688	547	327	874	19.2% (17.8%, 20.7%)	11.5% (10.3%, 12.7%)	15.4% (14.4%, 16.3%)
Li *et al.* 2005 (32)	2002	36570	33257	69827	1638	1239	2877	4.5% (4.3%, 4.7%)	3.7% (3.5%, 3.9%)	4.1% (4.0%, 4.3%)
Wang *et al.* 2005 (46)	2002	937	794	1731	122	107	229	13.0% (10.9%, 15.2%)	13.5% (11.1%, 15.9%)	13.2% (11.6%, 14.8%)
Zhang *et al.* 2003 (47)	2002	3099	2989	6088	226	211	437	7.3% (6.4%, 8.2%)	7.1% (6.2%, 8.0%)	7.2% (6.5%, 7.8%)
Zhang (b) *et al.* 2012 (58)	2003	35982	34449	70431	6140	3489	9629	17.1% (16.7%, 17.5%)	10.1% (9.8%, 10.4%)	13.7% (13.4%, 13.9%)
Ko *et al.* 2008 (59)	2003–2004	973	1104	2077	187	105	292	19.2% (16.7%, 21.7%)	9.5% (7.8%, 10.4%)	14.1% (12.6%, 15.6%)
Xiang *et al.* 2005 (49)	2004	12039	11253	23292	1740	1168	2908	14.5% (13.8%, 15.1%)	10.4% (9.8%, 10.9%)	12.5% (12.1%, 12.9%)
Shan *et al.* 2010 (50)	2004	10602	10596	21198	2452	1510	3962	23.1% (22.3%, 23.9%)	14.3% (13.6%, 14.9%)	18.7% (18.2%, 19.2%)
CHNS 2004 (29)	2004	770	693	1463	65	51	116	8.4% (6.5%, 10.4%)	7.4% (5.4%, 9.3%)	7.9% (6.5%, 9.3%)
CNSSCH 2007 (37)	2005	117570	116583	234153	12981	9805	22786	11.0% (10.9%, 11.2%)	8.4% (8.3%, 8.6%)	9.7% (9.6%, 9.9%)
**Sub-total**		**221390**	**214558**	**435948**	**26098**	**18012**	**44110**	**13.7% (10.1%, 17.3%)**	**9.5% (7.5%, 11.6%)**	**11.6% (8.8%, 14.5%)**
**2006–2010**										
CHNS 2006 (29)	2006	626	548	1174	63	35	98	10.1% (7.7%, 12.4%)	6.4% (4.3%, 8.4%)	8.3% (6.8%, 9.9%)
Wang *et al.* 2008 (51)	2006	9467	8853	18320	1351	1258	2609	14.3% (13.6%, 15.0%)	14.2% (13.5%, 14.9%)	14.2% (13.7%, 14.7%)
Lv *et al.* 2009 (52)	2006	3123	2893	6016	686	299	985	22.0% (20.5%, 23.4%)	10.3% (9.2%, 11.4%)	16.4% (15.4%, 17.3%)
Wang *et al.* 2011(53)	2006	5062	4922	9984	871	675	1546	17.2% (16.2%, 18.2%)	13.7% (12.8%, 14.7%)	15.5% (14.8%, 16.2%)
NTFCOC. 2008 (28)	2006	45139	39627	84766	10034	6750	16784	22.2% (21.8%, 22.6%)	17.0% (16.7%, 17.4%)	19.8% (19.5%, 20.1%)
Wu *et al.* 2008 (54)	2007	2209	1931	4140	272	132	404	12.3% (10.9%, 13.7%)	6.8% (5.7%, 8.0%)	9.8% (8.9%, 10.7%)
Zhang (b) *et al.* 2012 (58)	2008	44148	41457	85605	8307	4889	13196	18.8% (18.5%, 19.2%)	11.8% (11.5%, 12.1%)	15.4% (15.2%, 15.7%)
Ma *et al.* 2011 (60)	2008–2009	4498	4155	8653	488	462	950	10.8% (9.9%, 11.8%)	11.1% (10.2%, 12.1%)	11.0% (10.3%, 11.6%)
Chang *et al.* 2012 (55)	2009	7356	6638	13994	872	586	1458	11.9% (11.1%, 12.6%)	8.8% (8.1%, 9.5%)	10.4% (9.9%, 10.9%)
Cao *et al.* 2012 (56)	2009	44211	44763	88974	6467	3829	10296	14.6% (14.3%, 15.0%)	8.6% (8.3%, 8.8%)	11.6% (11.4%, 11.8%)
Andegiorgish *et al.* 2012(57)	2010	1559	1581	3140	234	160	394	15.0% (13.2%, 16.8%)	10.1% (8.6%, 11.6%)	12.5% (11.4%, 13.7%)
Wang *et al.*2012 (38)	2010	600	600	1200	83	66	149	13.8% (11.1%, 16.6%)	11.0% (8.5%, 13.5%)	12.4% (10.6%, 14.3%)
Liu *et al.*2012 (39)	2010	1200	1200	2400	180	84	264	15.0% (13.0%, 17.0%)	7.0% (5.6%, 8.4%)	11.0% (9.7%, 12.3%)
Zhang (a) *et al.*2012 (40)	2010	3783	3794	7577	656	454	1110	17.3% (16.1%, 18.5%)	12.0% (10.9%, 13.0%)	14.6% (13.9%, 15.4%)
**Sub-total**		**172981**	**162962**	**335943**	**30564**	**19679**	**50243**	**15.4% (13.3%, 17.5%)**	**10.7% (8.8%, 12.6%)**	**13.1% (11.2%, 15.0%)**
**Overall**		**1024710**	**991651**	**2016361**	**85452**	**64356**	**149808**	**11.5% (9.9%, 13.1%)**	**8.3% (7.2%, 9.3%)**	**9.9% (8.6%, 11.3%)**

Forty-one studies assessed the prevalence of obesity in a total of 2,225,347 Chinese children and adolescents (Table 2). A pooled analysis based on years of fieldwork divided into five-year periods demonstrated an increase in obesity over time (Table 2). The prevalence of obesity increased from 0.4% (95% CI, −0.1% to −0.8%) in all subjects, 0.4% (95% CI, −0.0% to 0.8%) in boys and 0.3% (95% CI, −0.2% to 0.8%) in girls in 1981–1985 to 7.5% (95% CI, 6.6%–8.4%) in all subjects, 9.3% (95% CI, 8.3%–10.4%) in boys and 5.3% (95% CI, 4.5%–6.2%) in girls in 2006–2010. These values represent 18.8-fold, 23.3-fold and 17.7-fold increases, respectively, since 1981–1985, with average annual increase rates of 12.4%, 13.4% and 12.2%, respectively.

**Table 2:** Summary of studies reporting the prevalence of obesity in Chinese children and adolescents aged 0–18 years.

Author, year	Time period	Sample size (n)			Obesity (n)			Obesity, Prevalence, % (95% CI)		
	(years)	Boys	Girls	Total	Boys	Girls	Total	Boys	Girls	Total
**1981–1985**										
Chen.1986 (30)	1982	5334	4793	10127	9	4	13	0.2% (0.1%, 0.3%)	0.1% (0.0%, 0.2%)	0.1% (0.1%, 0.2%)
CNSSCH 1987 (33)	1985	205100	204846	409946	1236	1186	2422	0.6% (0.6%, 0.6%)	0.6% (0.5%, 0.6%)	0.6% (0.6%, 0.6%)
**Sub-total**		**210434**	**209639**	**420073**	**1245**	**1190**	**2435**	**0.4% (−0.0%, 0.8%)**	**0.3% (−0.2%, 0.8%)**	**0.4% (−0.1%, 0.8%)**
**1986–1990**										
Ding *et al.* 1988 (41)	1986	4417	4503	8920	173	112	285	3.9% (3.3%, 4.5%)	2.5% (2.0%, 2.9%)	3.2% (2.8%, 3.6%)
Ding *et al.* 1989 (26)	1986	71420	66609	138029	576	490	1066	0.8% (0.7%, 0.9%)	0.7% (0.7%, 0.8%)	0.8% (0.7%, 0.8%)
**Sub-total**		**75837**	**71112**	**146949**	**749**	**602**	**1351**	**2.3% (−0.7%, 5.4%)**	**1.6% (−0.1%, 3.3%)**	**2.0% (−0.4%, 4.4%)**
**1991–1995**										
CHNS 1991 (29)	1991	1333	1248	2581	17	18	35	1.3% (0.7%, 1.9%)	1.4% (0.8%, 2.1%)	1.4% (0.9%, 1.8%)
CNSSCH 1993 (34)	1991	70608	70047	140655	1675	1097	2772	2.4% (2.3%, 2.5%)	1.6% (1.5%, 1.7%)	2.0% (1.9%, 2.0%)
Ge.1995 (31)	1992	13251	11987	25238	234	199	433	1.8% (1.5%, 2.0%)	1.7% (1.4%, 1.9%)	1.7% (1.6%, 1.9%)
CHNS 1993 (29)	1993	1248	1144	2392	20	21	41	1.6% (0.9%, 2.3%)	1.8% (1.1%, 2.6%)	1.7% (1.2%, 2.2%)
CNSSCH 1997 (35)	1995	103960	104676	208636	3919	2198	6117	3.8% (3.7%, 3.9%)	2.1% (2.0%, 2.2%)	2.9% (2.9%, 3.0%)
**Sub-total**		**190400**	**189102**	**379502**	**5865**	**3533**	**9398**	**2.2% (1.3%, 3.1%)**	**1.7% (1.4%, 2.1%)**	**2.0% (1.4%, 2.6%)**
**1996–2000**										
CHNS 1997 (29)	1997	1269	1120	2389	26	12	38	2.0% (1.3%, 2.8%)	1.1% (0.5%, 1.7%)	1.6% (1.1%, 2.1%)
CHNS 2000 (29)	2000	1216	1074	2290	23	14	37	1.9% (1.1%, 2.7%)	1.3% (0.6%, 2.0%)	1.6% (1.1%, 2.1%)
CNSSCH 2002 (36)	2000	111853	111919	223772	7348	3842	11190	6.6% (6.4%, 6.7%)	3.4% (3.3%, 3.5%)	5.0% (4.9%, 5.1%)
Zuo *et al.* 2000 (42)	1997	2108	1899	4007	192	80	272	9.1% (7.9%, 10.3%)	4.2% (3.3%, 5.1%)	6.8% (6.0%, 7.6%)
Hui *et al.* 2003 (43)	1999	2428	2146	4574	111	33	144	4.6% (3.7%, 5.4%)	1.5% (1.0%, 2.1%)	3.1% (2.6%, 3.7%)
Ding *et al.* 1998 (27)	1996	110993	97520	208513	2418	1858	4276	2.2% (2.1%, 2.3%)	1.9% (1.8%, 2.0%)	2.0% (2.0%, 2.1%)
Chen *et al.* 2002 (44)	2000	1746	1611	3357	139	98	237	8.0% (6.7%, 9.2%)	6.1% (4.9%, 7.3%)	7.1% (6.2%, 7.9%)
**Sub-total**		**231613**	**217289**	**448902**	**10257**	**5937**	**16194**	**4.9% (2.6%, 7.1%)**	**2.7% (1.8%, 3.5%)**	**3.9% (2.3%, 5.4%)**
**2001–2005**										
Xiang *et al.* 2005 (49)	2004	12039	11253	23292	601	263	864	5.0% (4.6%, 5.4%)	2.3% (2.1%, 2.6%)	3.7% (3.5%, 4.0%)
Shan *et al.* 2010 (50)	2004	10602	10596	21198	846	342	1188	8.0% (7.5%, 8.5%)	3.2% (2.9%, 3.6%)	5.6% (5.3%, 5.9%)
CHNS 2004 (29)	2004	770	693	1463	23	23	46	3.0% (1.8%, 4.2%)	3.3% (2.0%, 4.7%)	3.1% (2.2%, 4.0%)
CNSSCH 2007 (37)	2005	117570	116583	234153	10544	5129	15673	9.0% (8.8%, 9.1%)	4.4% (4.3%, 4.5%)	6.7% (6.6%, 6.8%)
Li *et al.* 2005 (32)	2002	36570	33257	69827	848	612	1460	2.3% (2.2%, 2.5%)	1.8% (1.7%, 2.0%)	2.1% (2.0%, 2.2%)
Li *et al.* 2007 (45)	2001	2848	2840	5688	132	48	180	4.6% (3.9%, 5.4%)	1.7% (1.2%, 2.2%)	3.2% (2.7%, 3.6%)
Zhang (b) *et al.* 2012 (58)	2003	35982	34449	70431	1914	706	2620	5.3% (5.1%, 5.6%)	2.0% (1.9%, 2.2%)	3.7% (3.6%, 3.9%)
Ko *et al.* 2008 (59)	2003–2004	973	1104	2077	54	41	95	5.5% (4.1%, 7.0%)	3.7% (2.6%, 4.8%)	4.6% (3.7%, 5.5%)
Wei *et al.* 2007 (48)	2001	30084	27946	58030	1621	1177	2798	5.4% (5.1%, 5.6%)	4.2% (4.0%, 4.4%)	4.8% (4.6%, 5.0%)
Wang *et al.* 2005 (46)	2002	937	794	1731	70	52	122	7.5% (5.8%, 9.2%)	6.5% (4.8%, 8.3%)	7.0% (5.8%, 8.3%)
Zhang *et al.* 2003 (47)	2002	3099	2989	6088	148	108	256	4.8% (4.0%, 5.5%)	3.6% (2.9%, 4.3%)	4.2% (3.7%, 4.7%)
**Sub-total**		**251474**	**242504**	**493978**	**16801**	**8501**	**25302**	**5.5% (3.7%, 7.3%)**	**3.3% (2.5%, 4.1%)**	**4.4% (3.2%, 5.7%)**
**2006–2010**										
CHNS 2006 (29)	2006	626	548	1174	33	25	58	5.3% (3.5%, 7.0%)	4.6% (2.8%, 6.3%)	4.9% (3.7%, 6.2%)
Wang *et al.* 2008 (51)	2006	9467	8853	18320	892	565	1457	9.4% (8.8%, 10.0%)	6.4% (5.9%, 6.9%)	8.0% (7.6%, 8.3%)
Lv *et al.* 2009 (52)	2006	3123	2893	6016	284	103	387	9.1% (8.1%, 10.1%)	3.6% (2.9%, 4.2%)	6.4% (5.8%, 7.1%)
Wu *et al.* 2008 (54)	2007	2209	1931	4140	207	99	306	9.4% (8.2%, 10.6%)	5.1% (4.1%, 6.1%)	7.4% (6.6%, 8.2%)
Zhang (b) *et al.* 2012 (58)	2008	44148	41457	85605	2909	1008	3917	6.6% (6.4%, 6.8%)	2.4% (2.3%, 2.6%)	4.6% (4.4%, 4.7%)
Chang *et al.* 2012 (55)	2009	7356	6638	13994	362	292	654	4.9% (4.4%, 5.4%)	4.4% (3.9%, 4.9%)	4.7% (4.3%, 5.0%)
Cao *et al.* 2012 (56)	2009	44211	44763	88974	3091	1282	4373	7.0% (6.8%, 7.2%)	2.9% (2.7%, 3.0%)	4.9% (4.8%, 5.1%)
Andegiorgish *et al.* 2012(57)	2010	1559	1581	3140	306	187	493	19.6% (17.7%, 21.6%)	11.8% (10.2%, 13.4%)	15.7% (14.4%, 17.0%)
Wang *et al.*2012 (38)	2010	600	600	1200	86	64	150	14.3% (11.5%, 17.1%)	10.7% (8.2%, 13.1%)	12.5% (10.6%, 14.4%)
Liu *et al.*2012 (39)	2010	1200	1200	2400	131	40	171	10.9% (9.2%, 12.7%)	3.3% (2.3%, 4.3%)	7.1% (6.1%, 8.2%)
Zhang (a) *et al.*2012 (40)	2010	3783	3794	7577	599	270	869	15.8% (14.7%, 17.0%)	7.1% (6.3%, 7.9%)	11.5% (10.8%, 12.2%)
Wang *et al.* 2011 (53)	2006	5062	4922	9984	354	203	557	7.0% (6.3%, 7.7%)	4.1% (3.6%, 4.7%)	5.6% (5.1%, 6.0%)
NTFCOC. 2008 (28)	2006	45139	39627	84766	4004	2086	6090	8.9% (8.6%, 9.1%)	5.3% (5.0%, 5.5%)	7.2% (7.0%, 7.4%)
Ma *et al.* 2011 (60)	2008–2009	4498	4155	8653	278	248	526	6.2% (5.5%, 6.9%)	6.0% (5.2%, 6.7%)	6.1% (5.6%, 6.6%)
**Sub-total**		**172981**	**162962**	**335943**	**13536**	**6472**	**20008**	**9.3% (8.3%, 10.4%)**	**5.3% (4.5%, 6.2%)**	**7.5% (6.6%, 8.4%)**
**Overall**		**1132739**	**1092608**	**2225347**	**48453**	**26235**	**74688**	**5.9% (5.1%, 6.6%)**	**3.4% (3.0%, 3.8%)**	**4.7% (4.1%, 5.3%)**

Further pooled analysis of the prevalence of overweight and obesity in Chinese children and adolescents, categorized according to years of fieldwork demonstrated an increase in overweight and obesity over time. The prevalence of both conditions increased from 2.1% (95% CI, 0.3%–4.0%) in all subjects, 1.9% (95% CI, 0.4%–3.5%) in boys and 2.4% (95% CI, 0.4%–4.4%) in girls in 1981–1985 to 20.7% (95% CI, 18.2%–23.1%) in all subjects, 25.0% (95% CI, 22.3%–27.7%) in boys and 16.2% (95% CI, 13.7%–18.6%) in girls in 2006–2010. These values represent 9.9-fold, 13.2-fold and 6.8-fold increases, respectively, since 1981–1985, with average annual increase rates of 12.1%, 13.8% and 10.0%, respectively.

### Gender differences

**Figure 2:**
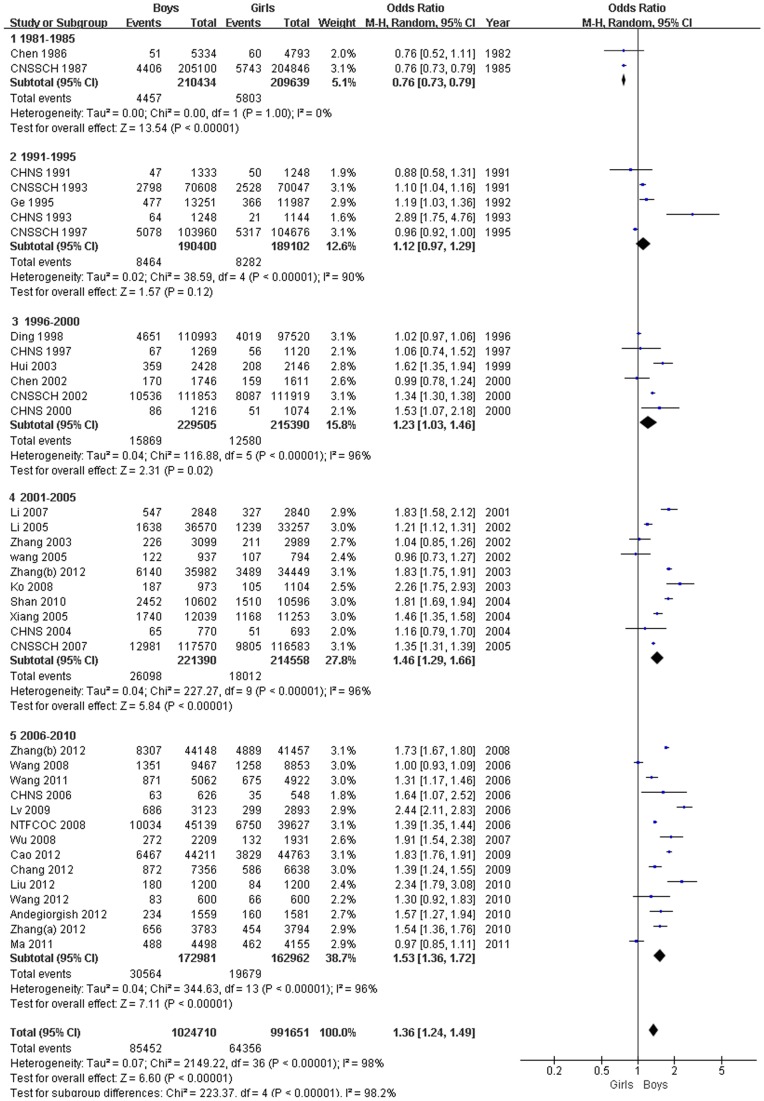
Forest plot of overweight in boys compared with girls (aged, 0–18 years).

A comparative meta-analysis of the 37 studies that assessed the prevalence of overweight showed that boys were more likely to be overweight than girls (OR, 1.36; 95% CI, 1.24–1.49; *P*<0.00001). Time trend analyses based on years of fieldwork showed that the OR of being overweight for boys compared with girls gradually increased from 0.76 (95% CI, 0.73–0.79) in 1981–1985 to 1.53 (95% CI, 1.36–1.52) in 2006–2010 (Figure 2).

**Figure 3:**
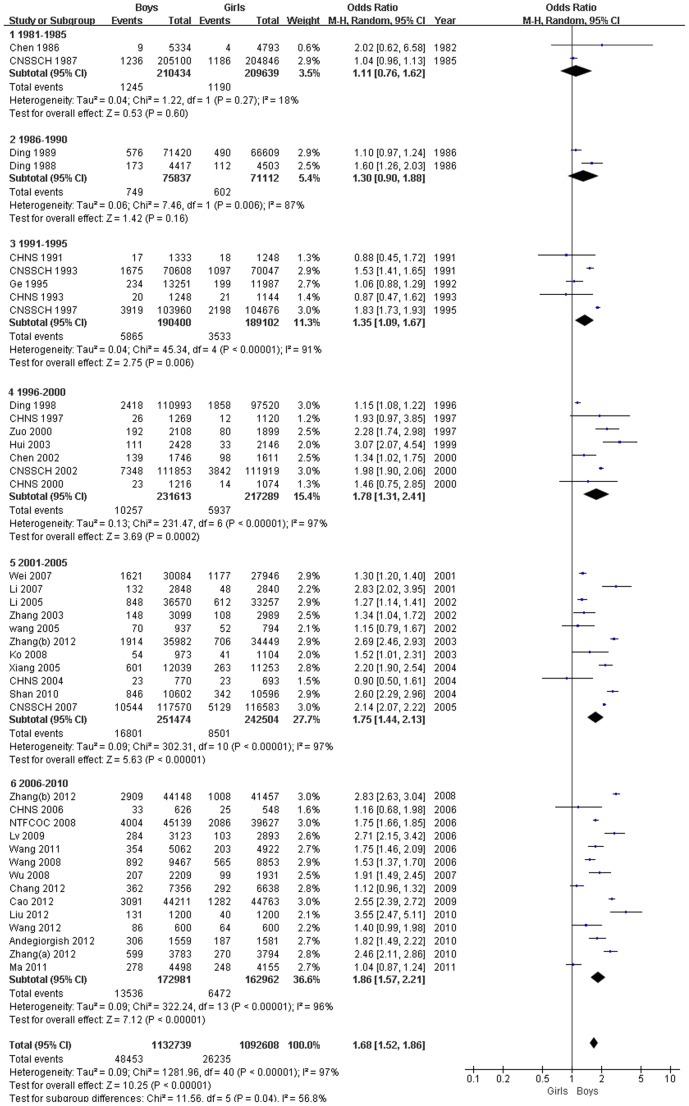
Forest plot of obesity in boys compared with girls (aged 0–18 years).

Moreover, a comparative meta-analysis of the 41 studies that assessed the prevalence of obesity showed that boys were more likely to be obese than girls (OR, 1.68; 95% CI, 1.52–1.86; *P*<0.00001). Time trend analyses based on years of fieldwork showed that the OR of being obese for boys compared with girls gradually increased from 1.11 (95% CI, 0.76–1.62) in 1981–1985 to 1.86 (95% CI, 1.57–2.21) in 2006–2010 (Figure 3).

### Urban-rural differences

Thirteen studies reported the prevalence of overweight/obesity among a total of 897,820 children and adolescents in urban and rural areas (Tables S2 and S3). In 2006–2010, the prevalence of overweight was 12.3% (95% CI, 8.1%–16.5%) in urban areas and 7.7% (95% CI, 6.5%–8.9%) in rural areas; these values were 2.3 and 2.1 times higher respectively than those in 1991–1995. The average annual increase rate was 5.6% and 5.2%, respectively (Table S2). In 2006–2010, the prevalence of obesity was 8.1% (95% CI, 5.4%–10.8%) in urban areas and 4.6% (95% CI, 3.7%–5.5%) in rural areas; these values were 2.8 and 3.5 times higher respectively than those in 1991–1995. The average annual increase rate was 7.1% and 8.8%, respectively (Table S3). With the exception of the prevalence of obesity in 1996–2000, in which there were no urban-rural differences (OR, 1.51; 95% CI, 0.71–3.22; *P* = 0.29), the prevalence of overweight/obesity in all other five-year periods from 1991–1995 to 2006–2010 was higher in urban areas than in rural areas (Figure S1, total OR for overweight, 1.66; 95% CI, 1.54–1.79; *P*<0.00001 and Figure S2, total OR for obesity, 1.97; 95% CI, 1.68–2.30; *P*<0.00001).

### Subgroup analysis

For subgroup analyses, the subjects were categorized according to growth and developmental stages (infancy, toddler, pre-school age, school age and adolescence; Table S4). The prevalence of overweight increased from 8.3% (95% CI, 8.0% to 8.6%) in infancy, 4.2% (95% CI, 4.0% to 4.3%) in toddler and 6.4% (95% CI, −0.2% to 13.0%) in pre-school age in 1996–2000 to 19.7% (95% CI, 19.0% to 20.5%) in infancy, 16.1% (95% CI, 9.6% to 22.6%) in toddler and 13.4% (95% CI, 9.2% to 17.7%) in pre-school age in 2006–2010, with average annual increase rates of 9.0%, 14.4% and 7.7%, respectively. The prevalence of overweight increased from 1.9% (95% CI, 1.2% to 2.6%) in school age and 1.8% (95% CI, −0.1% to 3.7%) in adolescence in 1981–1985 to 11.4% (95% CI, 9.4% to 13.4%) in school age and 11.4% (95% CI, 10.2% to 12.6%) in adolescence in 2006–2010, with average annual increase rates of 7.4% and 7.7%, respectively.

The prevalence of obesity increased from 1.7% (95% CI, 1.5% to 1.8%) in infancy, 0.4% (95% CI, 0.4% to 0.5%) in toddler and 0.7% (95% CI, 0.6% to 0.8%) in pre-school age in 1986–1990 to 6.0% (95% CI, 5.1% to 6.9%) in infancy, 6.0% (95% CI, 3.0% to 8.9%) in toddler and 6.8% (95% CI, 4.8% to 8.8%) in pre-school age in 2006–2010, with average annual increase rates of 6.5%, 14.5% and 12.0%, respectively. The prevalence of obesity increased from 0.5% (95% CI, 0.0% to 0.9%) in school age and 0.3% (95% CI, −0.2% to 0.7%) in adolescence in 1981–1985 to 9.0% (95% CI, 6.8% to 11.2%) in school age and 7.9% (95% CI, 5.6% to 10.3%) in adolescence in 2006–2010, with average annual increase rates of 12.3% and 14.0%, respectively. Both overweight and obesity increased more rapidly in the toddler stage than in other four stages (Tables 3 and S5).

**Table 3:** Subgroup analysis, by development stage, of the prevalence of overweight/obesity in Chinese children and adolescents aged 0–18 years.

	Infancy	Toddlers	Pre-school children	School children	Adolescents
**Overweight**					
1981–1985	-	-	-	1.9% (1.2%, 2.6%)	1.8% (−0.1%, 3.7%)
1991–1995	-	-	-	4.5% (3.4%, 5.6%)	3.5% (2.8%, 4.3%)
1996–2000	8.3% (8.0%, 8.6%)	4.2% (4.0%, 4.3%)	6.4% (−0.2%, 13.0%)	8.7% (6.6%, 10.8%)	5.3% (2.4%, 8.2%)
2001–2005	-	-	11.7% (10.5%, 12.9%)	14.2% (9.5%, 18.8%)	11.4% (7.5%, 15.3%)
2006–2010	19.7% (19.0%, 20.5%)	16.1% (9.6%, 22.6%)	13.4% (9.2%, 17.7%)	11.4% (9.4%, 13.4%)	11.4% (10.2%, 12.6%)
**Total**	**17.1% (9.6%, 24.5%)**	**13.7% (3.9%, 23.5%)**	**11.3% (6.2%, 16.5%)**	**9.1% (7.4%, 10.9%)**	**8.0% (6.6%, 9.5%)**
**Obesity**					
1981–1985	-	-	-	0.5% (0.0%, 0.9%)	0.3% (−0.2%, 0.7%)
1986–1990	1.7% (1.5%, 1.8%)	0.4% (0.4%, 0.5%)	0.7% (0.6%, 0.8%)	-	-
1991–1995	-	-	-	2.6% (2.0%, 3.2%)	1.3% (0.4%, 2.2%)
1996–2000	4.7% (4.5%, 5.0%)	1.4% (1.3%, 1.5%)	4.4% (−0.6%, 9.5%)	2.9% (1.2%, 4.7%)	3.5% (0.9%, 6.2%)
2001–2005	4.5% (4.1%, 5.0%)	2.1% (1.9%, 2.3%)	5.3% (3.4%, 7.3%)	5.6% (3.7%, 7.6%)	4.2% (2.8%, 5.5%)
2006–2010	6.0% (5.1%, 6.9%)	6.0% (3.0%, 8.9%)	6.8% (4.8%, 8.8%)	9.0% (6.8%, 11.2%)	7.9% (5.6%, 10.3%)
**Total**	**4.8% (2.9%, 6.7%)**	**3.8% (2.6%, 5.0%)**	**5.3% (3.9%, 6.7%)**	**5.0% (3.9%, 6.1%)**	**4.2% (3.4%, 5.1%)**

Subgroup analyses were also performed for urban-rural and sex-specific differences (Table S6). The results demonstrated that urban boys were more likely to be overweight and obese than urban girls, rural boys and rural girls, (total OR, 1.57; 95% CI, 1.55–1.60; *P*<0.00001; total OR, 2.07; 95% CI, 2.03–2.10; *P*<0.00001; and total OR, 2.52; 95% CI, 2.47–2.56; *P*<0.00001, respectively). The increase in the prevalence of overweight and obesity was the fastest in urban boys. The prevalence increased from 5.6% (95% CI, 5.1%–6.2%) for overweight, 3.3% (95% CI, 1.7%–5.0%) for obesity and 9.5% (95% CI, 7.4%–11.6%) for overweight and obesity in 1991–1995 to 16.0% (95% CI, 9.8%–22.1%) for overweight, 10.8% (95% CI, 4.4%–17.3%) for obesity and 26.6% (95% CI, 13.9%–39.2%) for overweight and obesity in 2006–2010. The 2006–2010 values are 2.9, 3.3 and 2.8 times higher than the corresponding values in 1991–1995, with an average annual increase rate of 7.2%, 8.2% and 7.1%, respectively.

### Analysis of heterogeneity and publication bias

Heterogeneity (*I* ^2^>50%) in pooled prevalence was high among the studies on overweight/obesity in children and adolescents. In 2006–2010, the prevalence of overweight ranged from 8.3% to 16.4%, and that of obesity, from 4.9% to 15.7%. Sensitivity analyses were performed (Appendix S5), and subgroups were based on difference in sample size, study quality, diagnostic criteria for overweight/obesity and geographical distribution. The results showed that all four factors contributed to the heterogeneity between the studies. Inspection of funnel plots did not reveal an obvious effect of publication bias, and the Egger test for publication bias was not statistically significant (*P* = 0.702 for studies assessing overweight in boys compared with girls, Appendix S6; *P* = 0.244 for studies assessing obesity in boys compared with girls, Appendix S7).

## Discussion

The present meta-analysis indicated that the prevalence of overweight/obesity has increased significantly among both boys and girls in China from 1981 to 2010. Similar trends were observed from infancy to adolescence and in urban and rural regions. Notably, the prevalence of overweight/obesity increased more rapidly in toddlers than the other growth and developmental stages. Additionally, the prevalence of overweight/obesity increased the fastest in urban boys. Sensitivity analyses found that difference in sample size, study quality, diagnostic criteria for overweight/obesity and geographical distribution all contributed to the heterogeneity between the studies. Funnel plots did not reveal an obvious effect of publication bias.

### Strengths and weaknesses

The problem of overweight/obesity in children and adolescents has attracted the attention of many Chinese researchers [61], and led to a number of epidemiological studies, including the four large national surveys and 23 regional studies assessed in this meta-analysis. National population-based surveys provide high quality data but are expensive and time-consuming [62]. Our analysis of these comprehensive studies describes the trend of overweight/obesity in children and adolescents in China in the past three decades and provides insightful data that can be used to inform decisions regarding policy and guide future scientific inquiry and experimentation.

Because of concerns about the quality of reporting in these studies and the possibility of publication bias, we attempted to detect publication bias by means of funnel plots and the Begg test. No signs of publication bias could be discovered in the funnel plot, and the results of the Begg test were not significant. This is the first meta-analysis to describe the trend in overweight/obesity in children and adolescents in China. The alarmingly rapid increase in the rate of overweight/obesity in children and adolescents warrants immediate intervention. Our results indicate that intervention strategies need to target high-risk groups, which include toddlers and urban boys.

This study has a number of potential limitations, and its findings should be interpreted with some caution. Like most meta-analyses, we pooled together studies conducted under different circumstances in different parts of China among different population groups. Moreover, some of the studies were not originally intended to report the prevalence of overweight/obesity in children and adolescents. There was therefore considerable heterogeneity in the studies combined. In addition, a specific limitation of our meta-analysis is related to the difficulty of combining studies that used four different diagnostic criteria to assess overweight/obesity. This is directly related to the lack of consensus about the diagnostic criteria for overweight/obesity. In order to evaluate the sources of bias in the review, we performed subgroup analyses (Table S6). The results showed that difference in sample size, study quality, diagnostic criteria for overweight/obesity and geographical distribution strongly influenced the prevalence of overweight and obesity among Chinese children and adolescents, and these factors may partially explain the between-studies heterogeneity.

The other variables tested did not further explain the heterogeneity. A number of well-conducted studies were not included in the pooled analysis, because they did not report separate results for boys and girls or urban and rural populations. The exclusion of these studies for these reasons may reduce the effectiveness of the results of our meta-analysis.

Moreover, all the studies included in our meta-analysis were of low or medium quality, except for the national surveys NSCO and CNSSCH, which were of high methodological quality. Therefore, an adequately powered, high-quality study is required to investigate the prevalence of overweight and obesity among Chinese children and adolescents.

Finally, various other factors may also have contributed to the prevalence of overweight and obesity among Chinese children and adolescents, such as demographic, behavioral, dietary, social and economic factors. Hence, further studies should adjust for these factors and analyze them at different levels.

### Explaining the findings

During the past three decades, China has experienced rapid socio-economic and nutritional transitions, which have led to a more obesogenic environment (e.g., increase in energy intake and decrease in physical activity) [63]. The traditional Chinese diet is shifting toward a diet with high fat, high energy density and low dietary fiber. Moreover, there has been a proliferation of fast food restaurants and an increase in activities that lead to reduced physical activity, such as watching television and playing computer games [64]. Television viewing besides reducing physical activity also promotes increased consumption of energy-rich foods through incessant commercial advertisements [65]. It strongly emphasizes on rote memorization and evaluates progress by a near-total reliance on test scores in China's education system, which lead to more reading, examinations or/and home works for Chinese children, less times for physical activities. These changes have resulted in a rapid increase in the prevalence of overweight/obesity in children and adolescents.

Sex differences in overweight/obesity were observed in the present study. Boys showed a higher prevalence of overweight/obesity than girls, which was consistent with the results of other Chinese studies [66]. Some studies in Western countries have also revealed that gender differences in the prevalence of overweight/obesity were common among children and adolescents [67], [68]. Boys and girls differ in body composition, patterns of weight gain, hormone biology and susceptibility to certain social, ethnic, genetic, and environmental factors, which led to the gender differences [69]. These differences in overweight/obesity trends may be related to the emerging social pressure of a preference for thinness among girls. Moreover, the traditional, societal preference for sons, particularly in rural areas, may mean that boys are likely to enjoy more of the family's resources [70]. The causes and consequences of overweight/obesity differ between the sexes. Thus, proposed interventions for these conditions should account for these differences.

Urban children and adolescents showed a higher prevalence of overweight/obesity than rural subjects, which was consistent with the results of other Chinese studies [71]. Economic development and changes in dietary patterns might explain these findings. Socioeconomic status (SES) has been proven to influence the prevalence of childhood overweight and obesity [72]. In China, urban residents have a higher SES than rural residents. National surveys have demonstrated a higher consumption of energy-dense, animal-based foods in children with a high SES, and a tendency for urban children to have a lower consumption of fruit and vegetables [73]. Compared with rural families, urban families own more televisions, video players and computers. It also seems probable that the increased use of automobiles, instead of bicycling or walking, in urban areas is a contributory factor [74]. Urban-rural differences in developed countries contradict the present results. The diet of high-SES groups in developed countries usually contains more vegetables and fruit than that of their low-SES peers. Thus, high-SES youths are less likely to be obese than their lower-SES counterparts [75]. Moreover, in developed countries, rural areas may offer limited opportunities for physical activity in children and adolescents, unlike urban areas, which have an abundance of parks and playgrounds [76]. Further subgroup analyses showed that urban boys were more likely to be overweight/obese than urban girls, rural boys and rural girls. Considering the abovementioned urban-rural and sex differences, urban boys face all the risk factors for overweight/obesity, making them the highest-risk population in the prevention and control of overweight/obesity.

### Implications for practice

In 2006–2010, the average annual prevalence of overweight, obesity, and both overweight and obesity in Chinese children and adolescents was lower than the corresponding prevalence for children and adolescents in the United States (16.5%, 16.9% and 31.8%, respectively) for the same period [77]. However, the average annual rates of increase in overweight and obesity from 1981 to 2010 were faster in China (6.9% and 12.8%, respectively) than in the United States (3.6% and 3.8%, respectively).

In the last 10 years, the prevalence of obesity/overweight among boys and girls has only slightly increased in the United States, from 14.0% and 13.8% in 1999–2000 to 18.6% and 15.0% in 2009–2010, giving average annual increase rates of 2.9% and 0.8% respectively [77]. In contrast, a rapid increase was seen in China in the same time frame.

Between 1980 and 2000 in the United States, the prevalence of overweight/obesity rapidly increased, which led to a growing public awareness of the epidemic [78]. State-level childhood obesity-prevention legislation intro duced since 2003 has achieved great success in preventing overweight/obesity in children and adolescents, and this may partially explain the slight increase in the prevalence of obesity/overweight in the United States in the past decade [79]. No such legislation yet exists in China; the Ministry of Health has issued guidelines for the prevention and control of overweight/obesity in Chinese children and adolescents, but these are rarely adopted in clinical practice [80]. Therefore, a requirement for the development of research policy and the introduction of legislation to prevent childhood obesity exists in China.

In the present study, overweight and obesity increased more rapidly in toddlers than in other growth and developmental stages. The toddler stage between 12 and 36 months of life is a critical period (when the child is transitioning from the all-milk diet of an infant to the family diet), and is also the period when children acquire many self-feeding skills [81]. In China, the one-child policy means that the child becomes the principal source of affection for two families (the husband's and the wife's). The child is spoiled and usually excessively fed because the parents worry too much about the child's growth and development [82]. Social and environmental influences also impact maternal feeding practices [83]. In China, many parents like to compare their baby's weight with those of other babies, mistakenly believing that heavier toddlers are healthier toddlers [84]. With the rapid development of the Chinese economy, parents have easier access to high fat, high energy dense foods than in the past, which might partially explain the rapid weight gain in toddlers. Toddlers learn from their parents about what to eat and why [85]. Parental modeling of eating habits can help shape children's values and beliefs related to food and eating behaviors [86]. Therefore, parents are given nutrition education to help toddlers develop healthy eating skills, which will improve the children's overall health and development and prevent obesity [87].

## Conclusions

The prevalence of overweight/obesity increased significantly among both boys and girls in China from 1981 to 2010. Similar trends were observed from infancy to adolescence and in urban and rural regions. Notably, the prevalence of overweight/obesity increased more rapidly in toddlers than in infants, pre-school children, school children and adolescents. Additionally, analysis of urban-rural and sex differences showed that the prevalence of overweight/obesity increased the fastest in urban boys. If this rapid growth persists, the increasing prevalence of overweight/obesity will be seriously detrimental to China's healthcare resources and produce significant increases in the economic costs of obesity and obesity-related illnesses. Public health prevention strategies are urgently needed to modify the health behaviors of children and adolescents in order to reduce the prevalence of overweight and obesity in China. Further national, population-based surveys on the prevalence of overweight/obesity in children and adolescents are required, and these should represent the entire population. Because overweight/obesity is affected by many factors, such as demographic, behavioral, dietary, social and economic factors, these factors should be analyzed and adjusted for in future surveys.

## Supporting Information

Checklist S1 **PRISMA Checklist for the meta-analysis.**
(DOC)

Figure S1 **Forest plot of overweight in urban children and adolescents compared with rural children and adolescents (ages, 0–18 years).**
(JPG)

Figure S2 **Forest plot of obesity in urban children and adolescents compared with rural children and adolescents (ages, 0–18 years).**
(JPG)

Table S1 **General description of studies reporting the prevalence of overweight and obesity in Chinese children and adolescents aged 0–18 years.**
(DOC)

Table S2 **Summary of studies reporting the urban and rural prevalence of overweight in children and adolescents aged 0–18 years.**
(DOC)

Table S3 **Summary of studies reporting the urban and rural prevalence of obesity in children and adolescents aged 0–18 years.**
(DOC)

Table S4 **Subgroup analysis, by sex and developmental stage, of the prevalence of overweight in Chinese children and adolescents aged 0–18 years.**
(DOC)

Table S5 **Subgroup analysis, by sex and developmental stage, of the prevalence of obesity in Chinese children and adolescents aged 0–18 years.**
(DOC)

Table S6 **Summary of studies reporting the prevalence of overweight/obesity in boys and girls in urban and rural areas.**
(DOC)

Appendix S1 **Search strategy for CNKI, Wanfang DATA, CINAHL, EMBASE and MEDLINE databases.**
(DOC)

Appendix S2 **Quality-assessment extraction form.**
(DOC)

Appendix S3 **General information on and study designs of the four included national surveys.**
(DOC)

Appendix S4 **Quality assessment (grade) of the 35 included papers (41 studies).**
(DOC)

Appendix S5 **Sensitivity analysis of the studies on the prevalence of overweight/obesity in children and adolescents.**
(DOC)

Appendix S6 **Funnel Plot and Begg test for meta-analysis of overweight in boys compared with girls (ages, 0–18 years).**
(DOC)

Appendix S7 **Funnel Plot and Begg test of for meta-analysis of obesity in boys compared with girls (ages, 0–18 years).**
(DOC)
