# Supplementary figures and images for: Trends in Overweight and Obesity among Children and Adolescents in China from 1981 to 2010: A Meta-Analysis
Source: PLoS One. 2012 Dec 17;7(12):e51949. doi: 10.1371/journal.pone.0051949 (PMC3524084; doi:10.1371/journal.pone.0051949)

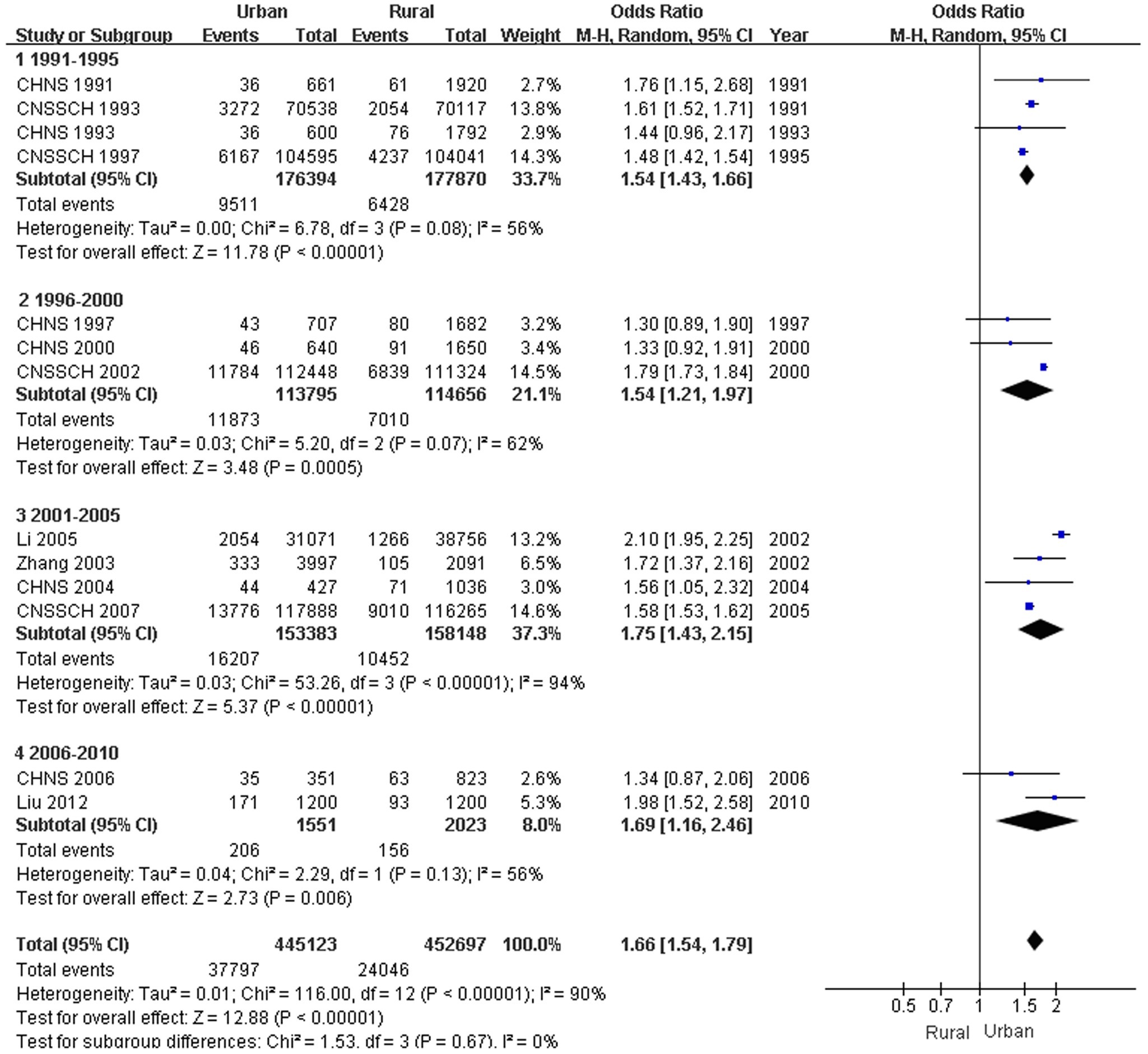

Supplement: Figure S1 — Forest plot of overweight in urban children and adolescents compared with rural children and adolescents (ages, 0–18 years). (JPG) [file pone.0051949.s002.jpg]

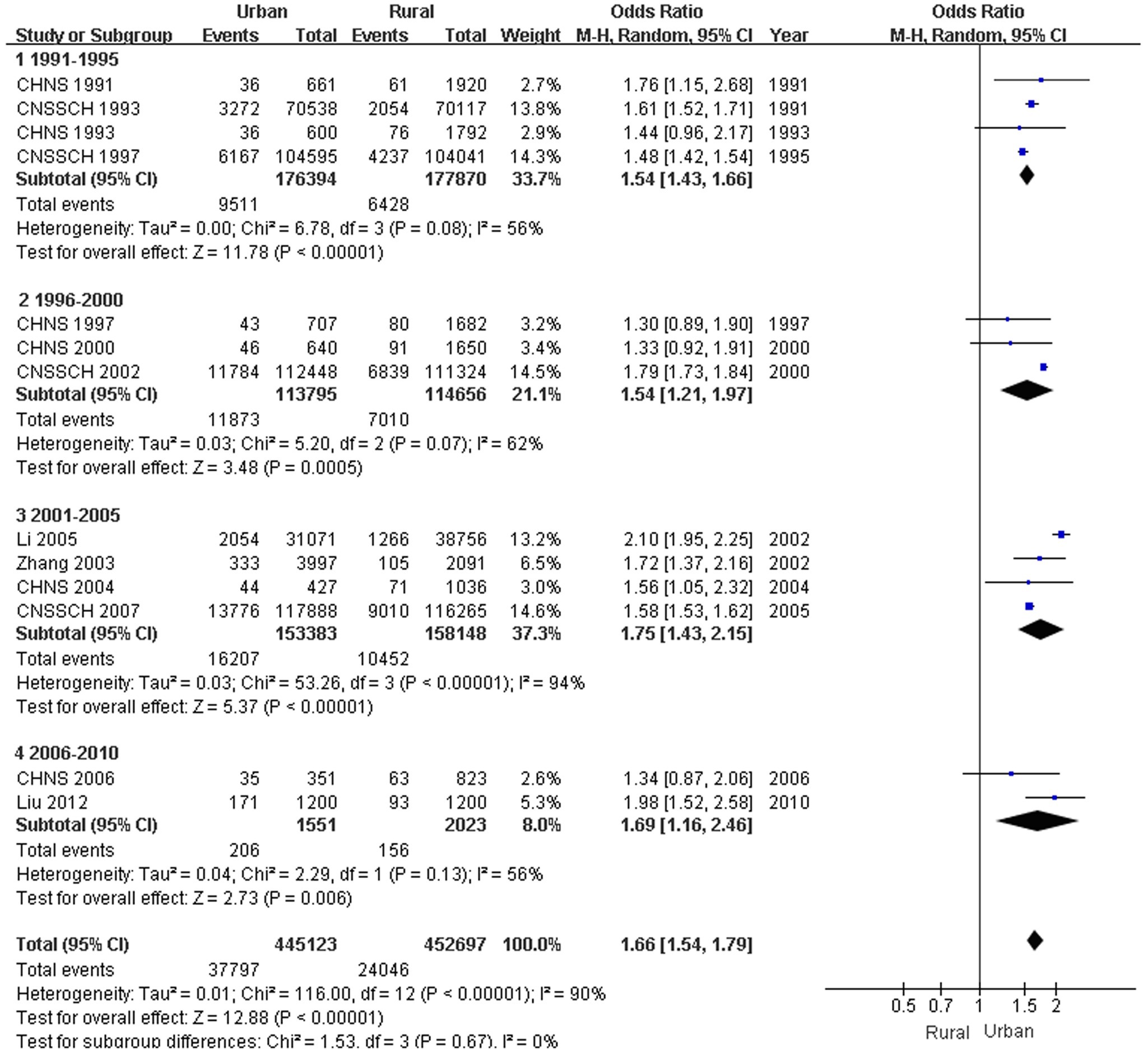

Supplement: Figure S2 — Forest plot of obesity in urban children and adolescents compared with rural children and adolescents (ages, 0–18 years). (JPG) [file pone.0051949.s003.jpg]
