# Supplementary material for: Trends in Overweight and Obesity among Children and Adolescents in China from 1981 to 2010: A Meta-Analysis
Source: PLoS One. 2012 Dec 17;7(12):e51949. doi: 10.1371/journal.pone.0051949 (PMC3524084; doi:10.1371/journal.pone.0051949)
Supplement: Table S1 — General description of studies reporting the prevalence of overweight and obesity in Chinese children and adolescents aged 0–18 years. (DOC) [file pone.0051949.s004.doc]

**Table S1.** General description of studies reporting the prevalence of overweight and obesity in Chinese children and adolescents aged 0–18 years

| **Author, year** | **Study objectives** | **Sampling frame and selection procedure** | **Time period** | **Sample size** | **Age (years)** | **Criteria** | **Target population** |
| --- | --- | --- | --- | --- | --- | --- | --- |
| **National Survey on Childhood Obesity** | | | | | | | |
| Ding *et al.* 1989 (26) | First National Survey on Childhood Obesity (1986) | National eight cities′ sample; two-stage stratified cluster sampling design | 1986 | 138029 | 0-7 | W/IW (WHO) | Urban children |
| Ding *et al.* 1998 (27) | Second National Survey on Childhood Obesity (1996) | National eleven cities′ sample; two-stage stratified cluster sampling design | 1996 | 208513 | 0-7 | W/IW (WHO) | Urban children |
| NTFCOC. 2008 (28) | Third National Survey on Childhood Obesity (2006) | National nine cities′ sample; two-stage stratified cluster sampling design | 2006 | 84766 | 0-7 | W/IW (WHO) | Urban children |
| **China Health and Nutrition Survey** | | | | | | | |
| CHNS (1991/1993/1997/  2000/2004/2006) (29) | China Health and Nutrition Survey (1991/1993/1997/  2000/2004/2006) | National seven provinces′ sample; multi-staged stratified cluster sampling design | 1991/1993/  1997/2000/  2004/2006 | 2581/2392/  2389/2290/  1463/1174 | 7-17 | BMI (IOTF) | Urban/rural |
| **Chinese national nutrition and health survey** | | | | | | | |
| Chen.1986 (30) | First Chinese National Nutrition and Health Survey (1982) | National 25 provinces and three municipalities′ sample; two-stage stratified cluster sampling design | 1982 | 10127 | 7-17 | BMI (IOTF) | Urban/rural |
| Ge.1995 (31) | Second Chinese National Nutrition and Health Survey (1992) | National 29 provinces and three municipalities′ sample; two-stage stratified cluster sampling design | 1992 | 25238 | 0-17 | BMI (IOTF) | Urban/rural |
| Li *et al.* 2005 (32) | Third Chinese National Nutrition and Health Survey (2002) | National 31 provinces, autonomous regions and the municipalities′ sample; two-stage stratified cluster sampling design | 2002 | 69827 | 0-17 | BMI (IOTF) | Urban/rural |
| **Chinese National Survey on Students Constitution and Health** | | | | | | | |
| CNSSCH 1987 (33) | First Chinese National Survey on Students Constitution and Health (1985) | National 30 provinces or municipalities′ sample; two-stage stratified cluster sampling design | 1985 | 409946 | 7-18 | W/IW (WHO) | Urban/rural |
| CNSSCH 1993 (34) | Second Chinese National Survey on Students Constitution and Health (1991) | National 30 provinces or municipalities′ sample; two-stage stratified cluster sampling design | 1991 | 140655 | 7-18 | W/IW (WHO) | upper socioeconomic classes |
| CNSSCH 1997 (35) | Third Chinese National Survey on Students Constitution and Health (1995) | National 30 provinces or municipalities′ sample; two-stage stratified cluster sampling design | 1995 | 208636 | 7-18 | W/IW (WHO) | Urban/rural |
| CNSSCH 2002 (36) | Fourth Chinese National Survey on Students Constitution and Health (2000) | National 30 provinces or municipalities′ sample; two-stage stratified cluster sampling design | 2000 | 223772 | 7-18 | W/IW (WHO) | Urban/rural |
| CNSSCH 2007 (37) | Fifth Chinese National Survey on Students Constitution and Health (2005) | National 30 provinces or municipalities′ sample; two-stage stratified cluster sampling design | 2005 | 234153 | 7-18 | W/IW (WHO) | Urban/rural |
| **Sixth Chinese National Survey on Students Constitution and Health (2010)** | | | | | | | |
| Wang *et al.*2012 (38) | Sixth Chinese National Survey on Students Constitution and Health (2010) | 8 schools randomly selected from Huairou district of Beijing city; two-stage stratified sampling | 2010 | 1200 | 7-18 | BMI (WGOC) | Not reported |
| Liu *et al.*2012 (39) | Sixth Chinese National Survey on Students Constitution and Health (2010) | Three urban schools, and four rural schools randomly selected from Urumqi city | 2010 | 2400 | 7-18 | BMI (WGOC) | Urban/rural |
| Zhang (a) *et al.*2012 (40) | Sixth Chinese National Survey on Students Constitution and Health (2010) | primary and secondary students randomly selected from three cities (Jinan, Yantai, Jining) of Shandong Province; two-stage stratified sampling | 2010 | 7577 | 7-18 | BMI (IOTF) | Not reported |
| **Regional Survey on Childhood Obesity** | | | | | | | |
| Ding *et al.* 1988 (41) | Assess obesity | 7 primary schools and 6 middle schools randomly selected from Beijing city; multi-staged stratified sampling | 1986 | 8920 | 7-18 | W/IW (WHO) | Urban |
| Zuo *et al.* 2000 (42) | Assess overweight and obesity | Three schools selected from Xicheng district of Xian city | 1997 | 4007 | 6-12 | W/IW (WHO) | Urban |
| Hui *et al.* 2003 (43) | Routine physical examinations in primary schools | Three schools randomly selected from Shenzhen city | 1999 | 4574 | 7-12 | BMI (IOTF) | Not reported |
| Chen *et al.* 2002 (44) | Assess overweight and obesity | 10 kindergartens randomly selected from Chaoyang district of Beijing city; multi-staged stratified sampling | 2000 | 3357 | 3-6 | W/IW (WHO) | Urban |
| Li *et al.* 2007 (45) | Assess overweight and obesity | primary and middle schools randomly selected from zhengzhou city; multi-staged stratified sampling | 2001 | 5688 | 7-18 | BMI (IOTF) | Urban |
| Wang *et al.* 2005 (46) | Assess overweight and obesity | 8 kindergartens randomly selected from three districts of Hefei city; multi-staged stratified sampling | 2002 | 1731 | 3-6 | W/IW (WHO) | Urban |
| Zhang *et al.* 2003 (47) | Assess overweight and obesity | 14 urban kindergartens, and 8 rural kindergartens randomly selected from Pudong district of Shanghai city; multi-staged stratified sampling | 2002 | 6088 | 1-7 | W/IW (WHO) | Urban/rural |
| Wei *et al.* 2007 (48) | Assess overweight and obesity | 19 districts or counties randomly selected from Shanghai city; multi-staged stratified sampling | 2002 | 58030 | 0-6 | W/IW (WHO) | Urban |
| Xiang *et al.* 2005 (49) | Assess overweight and obesity | 23 kindergartens, 25 primary and middle schools randomly selected from Chongqing city; multi-staged stratified sampling | 2004 | 23292 | 3-18 | BMI (IOTF) | Urban |
| Shan *et al.* 2010 (50) | Beijing Child and Adolescent Metabolic Syndromes Study | Four urban districts, and three suburb and rural districts randomly selected from Beijing city | 2004 | 21198 | 2-18 | BMI (IOTF) | Urban/rural |
| Wang *et al.* 2008 (51) | Assess overweight and obesity | 3 districts randomly selected from Chongqing city; multi-staged stratified sampling | 2006 | 18320 | 0-18 | BMI (CDC) | Urban |
| Lv *et al.* 2009 (52) | Assess overweight and obesity | 5 schools in Changzhou city; multi-staged stratified sampling | 2006 | 6016 | 7-12 | BMI (WGOC) | Urban |
| Wang *et al.* 2011 (53) | Assess overweight and obesity | kindergartens randomly selected from Zhoushan city; multi-staged stratified sampling | 2006 | 9984 | 0-6 | W/IW (WHO) | Not reported |
| Wu *et al.* 2008 (54) | Assess overweight and obesity | 5 schools in Changsha city; multi-staged stratified sampling | 2007 | 4140 | 7-12 | BMI (WGOC) | Not reported |
| Chang *et al.* 2012 (55) | Assess overweight and obesity | One urban district, and one sub-urban district randomly selected from Xian city | 2009 | 13994 | 7-18 | BMI (WGOC) | Urban and suburban |
| Cao *et al.* 2012 (56) | Assess obesity epidemical  situation and risk factors of childhood hypertension | 49 schools in three districts in Changsha city; multi-staged stratified sampling | 2009 | 88974 | 12-17 | BMI (WGOC) | Urban |
| Andegiorgish *et al.* 2012 (57) | Assess overweight and obesity | Five primary and middle schools randomly selected from Tianjin city; multi-staged stratified sampling | 2010 | 3140 | 12-15 | BMI (WGOC) | Urban/rural |
| Zhang (b) *et al.* 2012 (58) | Assess overweight and obesity | One urban district, and one suburb and rural district randomly selected from Shanghai city | 2003/2008 | 70431/85605 | 6-18 | BMI (IOTF) | Urban and suburban |
| Ko *et al.* 2008 (59) | Assess overweight and obesity according to various diagnostic criteria | 53 schools in Hong Kong; multi-staged stratified sampling | 2003-2004 | 2077 | 12-18 | BMI (IOTF) | Not reported |
| Ma *et al.* 2011 (60) | Nutrition Education in Urban Area of Northeast Chinese Children study | 48 kindergartens randomly selected from six cities (Benxi, Yingkou, Anshan, Fushun, Shenyang, Dalian) ; multi-staged stratified sampling | 2008-2009 | 8653 | 2-5 | BMI (IOTF) | urban |

BMI, body mass index; CDC, Centers for Disease Control and Prevention; IOTF, International Obesity Task Force; WGOC, Working Group for Obesity in China; WHO, World Health Organization; W/IW, ratio of weight (W) to ideal weight (IW); CNSSCH, Chinese National Survey on Students Constitution and Health; NTFCOC, National Task Force on Childhood Obesity of China.
