# Supplementary material for: Trends in Overweight and Obesity among Children and Adolescents in China from 1981 to 2010: A Meta-Analysis
Source: PLoS One. 2012 Dec 17;7(12):e51949. doi: 10.1371/journal.pone.0051949 (PMC3524084; doi:10.1371/journal.pone.0051949)
Supplement: Table S2 — Summary of studies reporting the urban and rural prevalence of overweight in children and adolescents aged 0–18 years. (DOC) [file pone.0051949.s005.doc]

**Table S2** Summary of studies and their reported prevalence of overweight in urban/rural children and adolescents age 0-18 years.

| Author, year | Time period | Sample size (n) | | | Overweight (n) | | | Overweight, Prevalence, % (95% CI) | | |
| --- | --- | --- | --- | --- | --- | --- | --- | --- | --- | --- |
|  | (years) | Urban | Rural | Total | Urban | Rural | Total | Urban | Rural | Total |
| **1991-1995** |  |  |  |  |  |  |  |  |  |  |
| CNSSCH 1993 (34) | 1991 | 70538 | 70117 | 140655 | 3272 | 2054 | 5326 | 4.6% (4.5%, 4.8%) | 2.9% (2.8%, 3.1%) | 3.8% (3.7%, 3.9%) |
| CHNS 1991 (29) | 1991 | 661 | 1920 | 2581 | 36 | 61 | 97 | 5.4% (3.7%, 7.2%) | 3.2% (2.4%, 4.0%) | 3.8% (3.0%, 4.5%) |
| CHNS 1993 (29) | 1993 | 600 | 1792 | 2392 | 36 | 76 | 112 | 6.0% (4.1%, 7.9%) | 4.2% (3.3%, 5.2%) | 4.7% (3.8%, 5.5%) |
| CNSSCH 1997 (35) | 1995 | 104595 | 104041 | 208636 | 6167 | 4237 | 10404 | 5.9% (5.8%, 6.0%) | 4.1% (4.0%, 4.2%) | 5.0% (4.9%, 5.1%) |
| **Sub-total** |  | 176394 | 177870 | 354264 | 9511 | 6428 | 15939 | 5.4% (4.4%, 6.4%) | 3.6% (2.8%, 4.4%) | 4.3% (3.4%, 5.2%) |
| **1996-2000** |  |  |  |  |  |  |  |  |  |  |
| CHNS 1997 (29) | 1997 | 707 | 1682 | 2389 | 43 | 80 | 123 | 6.1% (4.3%, 7.8%) | 4.8% (3.7%, 5.8%) | 5.1% (4.3%, 6.0%) |
| CHNS 2000 (29) | 2000 | 640 | 1650 | 2290 | 46 | 91 | 137 | 7.2% (5.2%, 9.2%) | 5.5% (4.4%, 6.6%) | 6.0% (5.0%, 7.0%) |
| CNSSCH 2002 (36) | 2000 | 112448 | 111324 | 223772 | 11784 | 6839 | 18623 | 10.5%(10.3%,10.7%) | 6.1% (6.0%, 6.3%) | 8.3% (8.2%, 8.4%) |
| **Sub-total** |  | 113795 | 114656 | 228451 | 11873 | 7010 | 18883 | 8.0% (4.8%, 11.2%) | 5.6% (4.7%, 6.5%) | 6.5% (4.3%, 8.8%) |
| **2001-2005** |  |  |  |  |  |  |  |  |  |  |
| Li *et al.* 2005 (32) | 2002 | 31071 | 38756 | 69827 | 2054 | 1266 | 3320 | 6.6% (6.3%, 6.9%) | 3.3% (3.1%, 3.4%) | 4.8% (4.6%, 4.9%) |
| Zhang *et al.* 2003 (47) | 2002 | 3997 | 2091 | 6088 | 333 | 105 | 438 | 8.3% (7.5%, 9.2%) | 5.0% (4.1%, 6.0%) | 7.2% (6.5%, 7.8%) |
| CHNS 2004 (29) | 2004 | 427 | 1036 | 1463 | 44 | 71 | 115 | 10.3% (7.4%, 13.2%) | 6.9% (5.3%, 8.4%) | 7.9% (6.5%, 9.2%) |
| CNSSCH 2007 (37) | 2005 | 117888 | 116265 | 234153 | 13776 | 9010 | 22786 | 11.7%(11.5%,11.9%) | 7.7% (7.6%, 7.9%) | 9.7% (9.6%, 9.9%) |
| **Sub-total** |  | 153383 | 158148 | 311531 | 16207 | 10452 | 26659 | 9.2% (5.8%, 12.6%) | 5.7% (2.7%, 8.8%) | 7.4% (4.0%, 10.7%) |
| **2006-2010** |  |  |  |  |  |  |  |  |  |  |
| CHNS 2006 (29) | 2006 | 351 | 823 | 1174 | 35 | 63 | 98 | 10.0% (6.8%, 13.1%) | 7.7% (5.8%, 9.5%) | 8.3% (6.8%, 9.9%) |
| Liu *et al.*2012 (39) | 2010 | 1200 | 1200 | 2400 | 171 | 93 | 264 | 14.2%(12.3%,16.2%) | 7.8% (6.2%, 9.3%) | 11.0% (9.7%, 12.3%) |
| **Sub-total** |  | 1551 | 2023 | 3574 | 206 | 156 | 362 | 12.3% (8.1%, 16.5%) | 7.7% (6.5%, 8.9%) | 9.7% (7.1%, 12.3%) |
| **Overall** |  | 445123 | 452697 | 897820 | 37797 | 24046 | 61843 | 8.2% (6.4%, 9.9%) | 5.3% (4.2%, 6.3%) | 6.6% (5.2%, 7.9%) |
