# Supplementary material for: Trends in Overweight and Obesity among Children and Adolescents in China from 1981 to 2010: A Meta-Analysis
Source: PLoS One. 2012 Dec 17;7(12):e51949. doi: 10.1371/journal.pone.0051949 (PMC3524084; doi:10.1371/journal.pone.0051949)
Supplement: Table S3 — Summary of studies reporting the urban and rural prevalence of obesity in children and adolescents aged 0–18 years. (DOC) [file pone.0051949.s006.doc]

**Table S3** Summary of studies and their reported prevalence of obesity in urban/rural children and adolescents age 0-18 years.

| Author, year | Time period | Sample size (n) | | | Obesity (n) | | | Obesity, Prevalence, % (95% CI) | | |
| --- | --- | --- | --- | --- | --- | --- | --- | --- | --- | --- |
|  | (years) | Urban | Rural | Total | Urban | Rural | Total | Urban | Rural | Total |
| **1991-1995** |  |  |  |  |  |  |  |  |  |  |
| CNSSCH 1993 (34) | 1991 | 70538 | 70117 | 140655 | 2133 | 639 | 2772 | 3.0% (2.9%, 3.2%) | 0.9% (0.8%, 1.0%) | 2.0% (1.9%, 2.0%) |
| CHNS 1991 (29) | 1991 | 661 | 1920 | 2581 | 10 | 25 | 35 | 1.5% (0.6%, 2.4%) | 1.3% (0.8%, 1.8%) | 1.4% (0.9%, 1.8%) |
| CHNS 1993 (29) | 1993 | 600 | 1792 | 2392 | 13 | 28 | 41 | 2.2% (1.0%, 3.3%) | 1.6% (1.0%, 2.1%) | 1.7% (1.2%, 2.2%) |
| CNSSCH 1997 (35) | 1995 | 104595 | 104041 | 208636 | 4653 | 1464 | 6117 | 4.4% (4.3%, 4.6%) | 1.4% (1.3%, 1.5%) | 2.9% (2.9%, 3.0%) |
| **Sub-total** |  | 176394 | 177870 | 354264 | 6809 | 2156 | 8965 | 2.9% (1.8%, 4.0%) | 1.3% (0.9%, 1.6%) | 2.0% (1.3%, 2.7%) |
| **1996-2000** |  |  |  |  |  |  |  |  |  |  |
| CHNS 1997 (29) | 1997 | 707 | 1682 | 2389 | 9 | 28 | 37 | 1.3% (0.4%, 2.1%) | 1.7% (1.1%, 2.3%) | 1.5% (1.1%, 2.0%) |
| CHNS 2000 (29) | 2000 | 640 | 1650 | 2290 | 13 | 24 | 37 | 2.0% (0.9%, 3.1%) | 1.5% (0.9%, 2.0%) | 1.6% (1.1%, 2.1%) |
| CNSSCH 2002 (36) | 2000 | 112448 | 111324 | 223772 | 7998 | 3202 | 11200 | 7.1% (7.0%,7.3%) | 2.9% (2.8%, 3.0%) | 5.0% (4.9%, 5.1%) |
| **Sub-total** |  | 113795 | 114656 | 228451 | 8020 | 3254 | 11274 | 3.5% (-0.01%, 7.9%) | 2.0% (1.0%, 3.1%) | 2.7% (0.0%, 5.5%) |
| **2001-2005** |  |  |  |  |  |  |  |  |  |  |
| Li *et al.* 2005 (32) | 2002 | 31071 | 38756 | 69827 | 1068 | 633 | 1701 | 3.4% (3.2%, 3.6%) | 1.6% (1.5%, 1.8%) | 2.4% (2.3%, 2.6%) |
| Zhang *et al.* 2003 (47) | 2002 | 3997 | 2091 | 6088 | 148 | 108 | 256 | 3.7% (3.1%, 4.3%) | 5.2% (4.2%, 6.1%) | 4.2% (3.7%, 4.7%) |
| CHNS 2004 (29) | 2004 | 427 | 1036 | 1463 | 18 | 28 | 46 | 4.2% (2.3%, 6.1%) | 2.7% (1.7%, 3.7%) | 3.1% (2.2%, 4.0%) |
| CNSSCH 2007 (37) | 2005 | 117888 | 116265 | 234153 | 10763 | 4910 | 15673 | 9.1% (9.0%, 9.3%) | 4.2% (4.1%, 4.3%) | 6.7% (6.6%, 6.8%) |
| **Sub-total** |  | 153383 | 158148 | 311531 | 11997 | 5679 | 17676 | 5.1% (1.3%, 8.9%) | 3.4% (1.6%, 5.2%) | 4.1% (1.3%, 7.0%) |
| **2006-2010** |  |  |  |  |  |  |  |  |  |  |
| CHNS 2006 (29) | 2006 | 351 | 823 | 1174 | 23 | 35 | 58 | 6.6% (4.0%, 9.1%) | 4.3% (2.9%, 5.6%) | 4.9% (3.7%, 6.2%) |
| Liu *et al.*2012 (39) | 2010 | 1200 | 1200 | 2400 | 112 | 59 | 171 | 9.3% (7.7%, 11.0%) | 4.9% (3.7%, 6.1%) | 7.1% (6.1%, 8.2%) |
| **Sub-total** |  | 1551 | 2023 | 3574 | 135 | 94 | 229 | 8.1% (5.4%, 10.8%) | 4.6% (3.7%, 5.5%) | 6.1% (3.9%, 8.2%) |
| **Overall** |  | 445123 | 452697 | 897820 | 26961 | 11183 | 38144 | 4.4% (3.0%, 5.8%) | 2.5% (1.8%, 3.2%) | 3.4% (2.4%, 4.4%) |
