# Supplementary material for: Trends in Overweight and Obesity among Children and Adolescents in China from 1981 to 2010: A Meta-Analysis
Source: PLoS One. 2012 Dec 17;7(12):e51949. doi: 10.1371/journal.pone.0051949 (PMC3524084; doi:10.1371/journal.pone.0051949)
Supplement: Table S4 — Subgroup analysis, by sex and developmental stage, of the prevalence of overweight in Chinese children and adolescents aged 0–18 years. (DOC) [file pone.0051949.s007.doc]

**Table S4** Subgroup analysis of the prevalence of overweight in Chinese children and adolescents age 0-18 years.

| Author, year | Time period | Sample size (n) | | | Overweight (n) | | | Overweight, Prevalence, % (95% CI) | | |
| --- | --- | --- | --- | --- | --- | --- | --- | --- | --- | --- |
|  | (years) | Boys | Girls | Total | Boys | Girls | Total | Boys | Girls | Total |
| **Infancy** |  |  |  |  |  |  |  |  |  |  |
| 1996-2000 |  |  |  |  |  |  |  |  |  |  |
| Ding *et al.* 1998 (27) | 1996 | 13629 | 12341 | 25970 | 1158 | 1001 | 2159 | 8.5% (8.0%, 9.0%) | 8.1% (7.6%, 8.6%) | 8.3% (8.0%, 8.6%) |
| Sub-total |  | 13629 | 12341 | 25970 | 1158 | 1001 | 2159 | 8.5% (8.0%, 9.0%) | 8.1% (7.6%, 8.6%) | 8.3% (8.0%, 8.6%) |
| 2006-2010 |  |  |  |  |  |  |  |  |  |  |
| Wang *et al.* 2008 (51) | 2006 | 2313 | 1979 | 4292 | 450 | 420 | 870 | 19.5% (17.8%, 21.1%) | 21.2% (19.4%, 23.0%) | 20.3% (19.1%, 21.5%) |
| Wang *et al.* 2011 (53) | 2006 | 967 | 889 | 1856 | 201 | 179 | 380 | 20.8% (18.2%, 23.3%) | 20.1% (17.5%, 22.8%) | 20.5% (18.6%, 22.3%) |
| NTFCOC. 2008 (28) | 2006 | 11455 | 9692 | 21147 | 2486 | 1599 | 4085 | 21.7% (20.9%, 22.5%) | 16.5% (15.8%, 17.2%) | 19.3% (18.8%, 19.8%) |
| Sub-total |  | 14735 | 12560 | 27295 | 3137 | 2198 | 5335 | 20.8% (19.2%, 22.3%) | 19.2% (15.7%, 22.7%) | 19.7% (19.0%, 20.5%) |
| **Overall** |  | **28364** | **24901** | **53265** | **4295** | **3199** | **7494** | **17.6% (9.1%, 26.1%)** | **16.4% (10.1%, 22.8%)** | **17.1% (9.6%, 24.5%)** |
| **Toddlers** |  |  |  |  |  |  |  |  |  |  |
| 1996-2000 |  |  |  |  |  |  |  |  |  |  |
| Ding *et al.* 1998 (27) | 1996 | 43680 | 38109 | 81789 | 1758 | 1656 | 3414 | 4.0% (3.8%, 4.2%) | 4.3% (4.1%, 4.6%) | 4.2% (4.0%, 4.3%) |
| Sub-total |  | 43680 | 38109 | 81789 | 1758 | 1656 | 3414 | 4.0% (3.8%, 4.2%) | 4.3% (4.1%, 4.6%) | 4.2% (4.0%, 4.3%) |
| 2006-2010 |  |  |  |  |  |  |  |  |  |  |
| Wang *et al.* 2008 (51) | 2006 | 1844 | 1741 | 3585 | 190 | 173 | 363 | 10.3% (8.9%, 11.7%) | 9.9% (8.5%, 11.3%) | 10.1% (9.1%, 11.1%) |
| Wang *et al.* 2011 (53) | 2006 | 2449 | 2383 | 4832 | 399 | 327 | 726 | 16.3% (14.8%, 17.8%) | 13.7% (12.3%, 15.1%) | 15.0% (14.0%, 16.0%) |
| NTFCOC. 2008 (28) | 2006 | 16698 | 15309 | 32007 | 4363 | 2880 | 7244 | 26.1% (25.5%, 26.8%) | 18.8% (18.2%, 19.4%) | 22.6% (22.2%, 23.1%) |
| Ma *et al.* 2011 (60) | 2008-2009 | 997 | 970 | 1967 | 184 | 144 | 328 | 18.5% (16.0%, 20.9%) | 14.8% (12.6%, 17.1%) | 16.7% (15.0%, 18.3%) |
| Sub-total |  | 21988 | 20403 | 42391 | 5136 | 3524 | 8661 | 17.8% (9.5%, 26.1%) | 14.3% (9.8%, 18.9%) | 16.1% (9.6%, 22.6%) |
| **Overall** |  | **65668** | **58512** | **124180** | **6894** | **5180** | **12075** | **15.0% (3.4%, 26.7%)** | **12.3% (4.5%, 20.2%)** | **13.7% (3.9%, 23.5%)** |
| **Pre-school children** |  |  |  |  |  |  |  |  |  |  |
| 1996-2000 |  |  |  |  |  |  |  |  |  |  |
| Ding *et al.* 1998 (27) | 1996 | 53684 | 47070 | 100754 | 1735 | 1362 | 3097 | 3.2% (3.1%, 3.4%) | 2.9% (2.7%, 3.0%) | 3.1% (3.0%, 3.2%) |
| Chen *et al.* 2002 (44) | 2000 | 1746 | 1611 | 3357 | 170 | 159 | 329 | 9.7% (8.3%, 11.1%) | 9.9% (8.4%, 11.3%) | 9.8% (8.8%, 10.8%) |
| Sub-total |  | 55430 | 48681 | 104111 | 1905 | 1521 | 3426 | 6.4% (0.1%, 12.8%) | 6.3% (-0.5%, 13.2%) | 6.4% (-0.2%, 13.0%) |
| 2001-2005 |  |  |  |  |  |  |  |  |  |  |
| Wang *et al.* 2005 (46) | 2002 | 937 | 794 | 1731 | 122 | 107 | 229 | 13.0% (10.9%, 15.2%) | 13.5% (11.1%, 15.9%) | 13.2% (11.6%, 14.8%) |
| Xiang *et al.* 2005 (49) | 2004 | 2794 | 2549 | 5343 | 342 | 259 | 601 | 12.2% (11.0%, 13.5%) | 10.2% (9.0%, 11.3%) | 11.2% (10.4%, 12.1%) |
| Shan *et al.* 2010 (50) | 2004 | 841 | 840 | 1681 | 89 | 97 | 186 | 10.6% (8.5%, 12.7%) | 11.5% (9.4%, 13.7%) | 11.1% (9.6%, 12.6%) |
| Sub-total |  | 4572 | 4183 | 8755 | 553 | 463 | 1016 | 12.0% (10.8%, 13.2%) | 11.5% (9.6%, 13.4%) | 11.7% (10.5%, 12.9%) |
| 2006-2010 |  |  |  |  |  |  |  |  |  |  |
| Wang *et al.* 2008 (51) | 2006 | 2141 | 2011 | 4152 | 283 | 292 | 575 | 13.2% (11.8%, 14.7%) | 14.5% (13.0%, 16.1%) | 13.8% (12.8%, 14.9%) |
| Wang *et al.* 2011 (53) | 2006 | 1646 | 1650 | 3296 | 271 | 169 | 440 | 16.5% (14.7%, 18.3%) | 10.2% (8.8%, 11.7%) | 13.3% (12.2%, 14.5%) |
| NTFCOC. 2008 (28) | 2006 | 16986 | 14626 | 31612 | 3185 | 2271 | 5455 | 18.8% (18.2%, 19.3%) | 15.5% (14.9%, 16.1%) | 17.3% (16.8%, 17.7%) |
| Ma *et al.* 2011 (60) | 2008-2009 | 3501 | 3185 | 6686 | 304 | 318 | 622 | 8.7% (7.8%, 9.6%) | 10.0% (8.9%, 11.0%) | 9.3% (8.6%, 10.0%) |
| Sub-total |  | 24274 | 21472 | 45746 | 4043 | 3050 | 7092 | 14.3% (8.8%, 19.8%) | 12.6% (9.4%, 15.8%) | 13.4% (9.2%, 17.7%) |
| **Overall** |  | **84276** | **74336** | **158612** | **6501** | **5034** | **11534** | **11.8% (6.3%, 17.3%)** | **10.9% (6.2%, 15.6%)** | **11.3% (6.2%, 16.5%)** |
| **School children** |  |  |  |  |  |  |  |  |  |  |
| 1981-1985 |  |  |  |  |  |  |  |  |  |  |
| Chen.1986 (30) | 1982 | 1985 | 1978 | 3963 | 34 | 26 | 60 | 1.7% (1.1%, 2.3%) | 1.3% (0.8%, 1.8%) | 1.5% (1.1%, 1.9%) |
| CNSSCH 1987 (33) | 1985 | 102696 | 102697 | 205393 | 2049 | 2474 | 4523 | 2.0% (1.9%, 2.1%) | 2.4% (2.3%, 2.5%) | 2.2% (2.1%, 2.3%) |
| Sub-total |  | 104681 | 104675 | 209356 | 2083 | 2500 | 4583 | 2.0% (1.9%, 2.1%) | 1.9% (0.8%, 3.0%) | 1.9% (1.2%, 2.6%) |
| 1991-1995 |  |  |  |  |  |  |  |  |  |  |
| CHNS 1991 (29) | 1991 | 771 | 696 | 1467 | 31 | 30 | 61 | 4.0% (2.6%, 5.4%) | 4.3% (2.8%, 5.8%) | 4.2% (3.1%, 5.2%) |
| CNSSCH 1993 (34) | 1991 | 35275 | 35030 | 70305 | 1382 | 1212 | 2594 | 3.9% (3.7%, 4.1%) | 3.5% (3.3%, 3.7%) | 3.7% (3.6%, 3.8%) |
| CHNS 1993 (29) | 1993 | 748 | 657 | 1405 | 49 | 19 | 68 | 6.6% (4.8%, 8.3%) | 2.9% (1.6%, 4.2%) | 4.8% (3.7%, 6.0%) |
| CNSSCH 1997 (35) | 1995 | 52371 | 52540 | 104911 | 2743 | 2773 | 5516 | 5.2% (5.0%, 5.4%) | 5.3% (5.1%, 5.5%) | 5.3% (5.1%, 5.4%) |
| Sub-total |  | 89165 | 88923 | 178088 | 4205 | 4034 | 8239 | 4.8% (3.8%, 5.8%) | 4.0% (2.7%, 5.3%) | 4.5% (3.4%, 5.6%) |
| 1996-2000 |  |  |  |  |  |  |  |  |  |  |
| CHNS 1997 (29) | 1997 | 793 | 684 | 1477 | 51 | 43 | 94 | 6.4% (4.7%, 8.1%) | 6.3% (4.5%, 8.1%) | 6.4% (5.1%, 7.6%) |
| CHNS 2000 (29) | 2000 | 674 | 586 | 1260 | 49 | 35 | 84 | 7.3% (5.3%, 9.2%) | 6.0% (4.1%, 7.9%) | 6.7% (5.3%, 8.0%) |
| CNSSCH 2002 (36) | 2000 | 56106 | 55990 | 112096 | 5680 | 4561 | 10241 | 10.1% (9.9%, 10.4%) | 8.1% (7.9%, 8.4%) | 9.1% (9.0%, 9.3%) |
| Hui *et al.* 2003 (43) | 1999 | 2428 | 2146 | 4574 | 359 | 208 | 567 | 14.8% (13.4%, 16.2%) | 9.7% (8.4%, 10.9%) | 12.4% (11.4%, 13.4%) |
| Sub-total |  | 60001 | 59406 | 119407 | 6139 | 4847 | 10986 | 9.7% (6.8%, 12.6%) | 7.7% (6.4%, 9.1%) | 8.7% (6.6%, 10.8%) |
| 2001-2005 |  |  |  |  |  |  |  |  |  |  |
| Xiang *et al.* 2005 (49) | 2004 | 4588 | 4063 | 8651 | 705 | 498 | 1203 | 15.4% (14.3%, 16.4%) | 12.3% (11.2%, 13.3%) | 13.9% (13.2%, 14.6%) |
| Shan *et al.* 2010 (50) | 2004 | 5533 | 5254 | 10787 | 1407 | 852 | 2259 | 25.4% (24.3%, 26.6%) | 16.2% (15.2%, 17.2%) | 20.9% (20.2%, 21.7%) |
| CHNS 2004 (29) | 2004 | 376 | 361 | 737 | 39 | 37 | 76 | 10.4% (7.3%, 13.5%) | 10.2% (7.1%, 13.4%) | 10.3% (8.1%, 12.5%) |
| CNSSCH 2007 (37) | 2005 | 58667 | 57848 | 116515 | 7533 | 5696 | 13229 | 12.8% (12.6%, 13.1%) | 9.8% (9.6%, 10.1%) | 11.4% (11.2%, 11.5%) |
| Sub-total |  | 69164 | 67526 | 136690 | 9684 | 7083 | 16767 | 16.1% (10.1%, 22.0%) | 12.2% (8.9%, 15.5%) | 14.2% (9.5%, 18.8%) |
| 2006-2010 |  |  |  |  |  |  |  |  |  |  |
| CHNS 2006 (29) | 2006 | 371 | 319 | 690 | 40 | 28 | 68 | 10.8% (7.6%, 13.9%) | 8.8% (5.7%, 11.9%) | 9.9% (7.6%, 12.1%) |
| Wang *et al.* 2008 (51) | 2006 | 1587 | 1479 | 3066 | 236 | 136 | 372 | 14.9% (13.1%, 16.6%) | 9.2% (7.7%, 10.7%) | 12.1% (11.0%, 13.3%) |
| Lv *et al.* 2009 (52) | 2006 | 3123 | 2893 | 6016 | 686 | 299 | 985 | 22.0% (20.5%, 23.4%) | 10.3% (9.2%, 11.4%) | 16.4% (15.4%, 17.3%) |
| Wu *et al.* 2008 (54) | 2007 | 2209 | 1931 | 4140 | 272 | 132 | 404 | 12.3% (10.9%, 13.7%) | 6.8% (5.7%, 8.0%) | 9.8% (8.9%, 10.7%) |
| Chang *et al.* 2012 (55) | 2009 | 3664 | 2860 | 6524 | 486 | 240 | 726 | 13.3% (12.2%, 14.4%) | 8.4% (7.4%, 9.4%) | 11.1% (10.4%, 11.9%) |
| Wang *et al.*2012 (38) | 2010 | 300 | 300 | 600 | 34 | 27 | 61 | 11.3% (7.7%, 14.9%) | 9.0% (5.8%, 12.2%) | 10.2% (7.7%, 12.6%) |
| Liu *et al.*2012 (39) | 2010 | 600 | 600 | 1200 | 85 | 34 | 119 | 14.2% (11.4%, 17.0%) | 5.7% (3.8%, 7.5%) | 9.9% (8.2%, 11.6%) |
| Sub-total | 11854 | 10382 | 22236 | 1839 | 896 | 2735 | 11854 | 14.2% (11.1%, 17.3%) | 8.3% (7.0%, 9.6%) | 11.4% (9.4%, 13.4%) |
| **Overall** | **334865** | **330912** | **665777** | **23950** | **19360** | **43310** | **334865** | **10.7% (8.6%, 12.7%)** | **7.4% (6.0%, 8.9%)** | **9.1% (7.4%, 10.9%)** |
| **Adolescents** |  |  |  |  |  |  |  |  |  |  |
| 1981-1985 |  |  |  |  |  |  |  |  |  |  |
| Chen.1986 (30) | 1982 | 3349 | 2815 | 6164 | 17 | 34 | 51 | 0.5% (0.3%, 0.7%) | 1.2% (0.8%, 1.6%) | 0.8% (0.6%, 1.1%) |
| CNSSCH 1987 (33) | 1985 | 102404 | 102149 | 204553 | 2357 | 3269 | 5626 | 2.3% (2.2%, 2.4%) | 3.2% (3.1%, 3.3%) | 2.8% (2.7%, 2.8%) |
| Sub-total |  | 105753 | 104964 | 210717 | 2374 | 3303 | 5677 | 1.4% (-0.3%, 3.2%) | 2.2% (0.3%, 4.2%) | 1.8% (-0.1%, 3.7%) |
| 1991-1995 |  |  |  |  |  |  |  |  |  |  |
| CHNS 1991 (29) | 1991 | 562 | 552 | 1114 | 16 | 20 | 36 | 2.8% (1.5%, 4.2%) | 3.6% (2.1%, 5.2%) | 3.2% (2.2%, 4.3%) |
| CNSSCH 1993 (34) | 1991 | 35333 | 35017 | 70350 | 1416 | 1316 | 2732 | 4.0% (3.8%, 4.2%) | 3.8% (3.6%, 4.0%) | 3.9% (3.7%, 4.0%) |
| CHNS 1993 (29) | 1993 | 500 | 487 | 987 | 15 | 2 | 17 | 3.0% (1.5%, 4.5%) | 0.4% (-0.2%, 1.0%) | 1.7% (0.9%, 2.5%) |
| CNSSCH 1997 (35) | 1995 | 51589 | 52136 | 103725 | 2335 | 2544 | 4879 | 4.5% (4.3%, 4.7%) | 4.9% (4.7%, 5.1%) | 4.7% (4.6%, 4.8%) |
| Sub-total |  | 87984 | 88192 | 176176 | 3782 | 3882 | 7664 | 3.0% (3.5%, 4.5%) | 3.2% (1.7%, 4.6%) | 3.5% (2.8%, 4.3%) |
| 1996-2000 |  |  |  |  |  |  |  |  |  |  |
| CHNS 1997 (29) | 1997 | 476 | 436 | 912 | 16 | 13 | 29 | 3.4% (1.7%, 5.0%) | 3.0% (1.4%, 4.6%) | 3.2% (2.0%, 4.3%) |
| CHNS 2000 (29) | 2000 | 542 | 488 | 1030 | 37 | 16 | 53 | 6.8% (4.7%, 8.9%) | 3.3% (1.7%, 4.9%) | 5.1% (3.8%, 6.5%) |
| CNSSCH 2002 (36) | 2000 | 55747 | 55929 | 111676 | 4856 | 3526 | 8382 | 8.7% (8.5%, 8.9%) | 6.3% (6.1%, 6.5%) | 7.5% (7.4%, 7.7%) |
| Sub-total |  | 56765 | 56853 | 113618 | 4909 | 3555 | 8464 | 6.3% (2.8%, 9.9%) | 4.3% (1.7%, 6.8%) | 5.3% (2.4%, 8.2%) |
| 2001-2005 |  |  |  |  |  |  |  |  |  |  |
| Xiang *et al.* 2005 (49) | 2004 | 4657 | 4641 | 9298 | 693 | 411 | 1104 | 14.9% (13.9%, 15.9%) | 8.9% (8.0%, 9.7%) | 11.9% (11.2%, 12.5%) |
| Shan *et al.* 2010 (50) | 2004 | 4228 | 4502 | 8730 | 956 | 561 | 1517 | 22.6% (21.4%, 23.9%) | 12.5% (11.5%, 13.4%) | 17.4% (16.6%, 18.2%) |
| CHNS 2004 (29) | 2004 | 394 | 332 | 726 | 26 | 14 | 40 | 6.6% (4.1%, 9.1%) | 4.2% (2.1%, 6.4%) | 5.5% (3.8%, 7.2%) |
| CNSSCH 2007 (37) | 2005 | 58903 | 58735 | 117638 | 5448 | 4109 | 9557 | 9.2% (9.0%, 9.5%) | 7.0% (6.8%, 7.2%) | 8.1% (8.0%, 8.3%) |
| Ko *et al.* 2008 (59) | 2003-2004 | 973 | 1104 | 2077 | 187 | 105 | 292 | 19.2% (16.7%, 21.7%) | 9.5% (7.8%, 11.2%) | 14.1% (12.6%, 15.6%) |
| Sub-total |  | 69155 | 69314 | 138469 | 7310 | 5200 | 12510 | 14.5% (8.8%, 20.2%) | 8.5% (6.2%, 10.8%) | 11.4% (7.5%, 15.3%) |
| 2006-2010 |  |  |  |  |  |  |  |  |  |  |
| CHNS 2006 (29) | 2006 | 255 | 229 | 484 | 23 | 7 | 30 | 9.0% (5.5%, 12.5%) | 3.1% (0.8%, 5.3%) | 6.2% (4.1%, 8.3%) |
| Wang *et al.* 2008 (51) | 2006 | 1582 | 1643 | 3225 | 192 | 237 | 429 | 12.1% (10.5%, 13.7%) | 14.4% (12.7%, 16.1%) | 13.3% (12.1%, 14.5%) |
| Chang *et al.* 2012 (55) | 2009 | 3692 | 3778 | 7470 | 386 | 346 | 732 | 10.5% (9.5%, 11.4%) | 9.2% (8.2%, 10.1%) | 9.8% (9.1%, 10.5%) |
| Cao *et al.* 2012 (56) | 2009 | 44211 | 44763 | 88974 | 6467 | 3829 | 10296 | 14.6% (14.3%, 15.0%) | 8.6% (8.3%, 8.8%) | 11.6% (11.4%, 11.8%) |
| Andegiorgish *et al.*2012 (57) | 2010 | 1559 | 1581 | 3140 | 234 | 160 | 394 | 15.0% (13.2%, 16.8%) | 10.1% (8.6%, 11.6%) | 12.5% (11.4%, 13.7%) |
| Wang *et al.*2012 (38) | 2010 | 300 | 300 | 600 | 49 | 39 | 88 | 16.3% (12.2%, 20.5%) | 13.0% (9.2%, 16.8%) | 14.7% (11.8%, 17.5%) |
| Liu *et al.*2012 (39) | 2010 | 600 | 600 | 1200 | 95 | 50 | 145 | 15.8% (12.9%, 18.8%) | 8.3% (6.1%, 10.5%) | 12.1% (10.2%, 13.9%) |
| Sub-total |  | 52199 | 52894 | 105093 | 7446 | 4668 | 12114 | 13.3% (11.3%, 15.2%) | 9.4% (7.7%, 11.1%) | 11.4% (10.2%, 12.6%) |
| **Overall** |  | **371856** | **372217** | **744073** | **25821** | **20608** | **46429** | **9.5% (7.7%, 11.3%)** | **6.4% (5.4%, 7.5%)** | **8.0% (6.6%, 9.5%)** |
