# Supplementary material for: Trends in Overweight and Obesity among Children and Adolescents in China from 1981 to 2010: A Meta-Analysis
Source: PLoS One. 2012 Dec 17;7(12):e51949. doi: 10.1371/journal.pone.0051949 (PMC3524084; doi:10.1371/journal.pone.0051949)
Supplement: Table S5 — Subgroup analysis, by sex and developmental stage, of the prevalence of obesity in Chinese children and adolescents aged 0–18 years. (DOC) [file pone.0051949.s008.doc]

**Table S5** Subgroup analysis of the prevalence of obesity in Chinese children and adolescents age 0-18 years.

| Author, year | Time period | Sample size (n) | | | Obesity (n) | | | Obesity, Prevalence, % (95% CI) | | |
| --- | --- | --- | --- | --- | --- | --- | --- | --- | --- | --- |
|  | (years) | Boys | Girls | Total | Boys | Girls | Total | Boys | Girls | Total |
| **Infancy** |  |  |  |  |  |  |  |  |  |  |
| 1986-1990 |  |  |  |  |  |  |  |  |  |  |
| Ding *et al.* 1989 (26) | 1986 | 14549 | 13056 | 27605 | 269 | 187 | 456 | 1.8% (1.6%, 2.1%) | 1.4% (1.2%, 1.6%) | 1.7% (1.5%, 1.8%) |
| Sub-total |  | 14549 | 13056 | 27605 | 269 | 187 | 456 | 1.8% (1.6%, 2.1%) | 1.4% (1.2%, 1.6%) | 1.7% (1.5%, 1.8%) |
| 1996-2000 |  |  |  |  |  |  |  |  |  |  |
| Ding *et al.* 1998 (27) | 1996 | 13629 | 12341 | 25970 | 675 | 550 | 1225 | 5.0% (4.6%, 5.3%) | 4.5% (4.1%, 4.8%) | 4.7% (4.5%, 5.0%) |
| Sub-total |  | 13629 | 12341 | 25970 | 675 | 550 | 1225 | 5.0% (4.6%, 5.3%) | 4.5% (4.1%, 4.8%) | 4.7% (4.5%, 5.0%) |
| 2001-2005 |  |  |  |  |  |  |  |  |  |  |
| Wei *et al.* 2007 (48) | 2001 | 3778 | 3484 | 7262 | 192 | 137 | 329 | 5.1% (4.4%, 5.8%) | 3.9% (3.3%, 4.6%) | 4.5% (4.1%, 5.0%) |
| Sub-total |  | 3778 | 3484 | 7262 | 192 | 137 | 329 | 5.1% (4.4%, 5.8%) | 3.9% (3.3%, 4.6%) | 4.5% (4.1%, 5.0%) |
| 2006-2010 |  |  |  |  |  |  |  |  |  |  |
| Wang *et al.* 2008(51) | 2006 | 2313 | 1979 | 4292 | 126 | 94 | 220 | 5.4% (4.5%, 6.4%) | 4.7% (3.8%, 5.7%) | 5.1% (4.5%, 5.8%) |
| Wang *et al.* 2011(53) | 2006 | 967 | 889 | 1856 | 80 | 44 | 124 | 8.3% (6.5%, 10.0%) | 4.9% (3.5%, 6.4%) | 6.7% (5.5%, 7.8%) |
| NTFCOC. 2008 (28) | 2006 | 11455 | 9692 | 21147 | 939 | 407 | 1346 | 8.2% (7.7%, 8.7%) | 4.2% (3.8%, 4.6%) | 6.4% (6.0%, 6.7%) |
| Sub-total |  | 14735 | 12560 | 27295 | 1145 | 545 | 1690 | 7.3% (5.3%, 9.3%) | 4.3% (4.0%, 4.7%) | 6.0% (5.1%, 6.9%) |
| **Overall** |  | **46691** | **41441** | **88132** | **2281** | **1419** | **3700** | **5.6% (3.3%, 7.9%)** | **3.9% (2.4%, 5.4%)** | **4.8% (2.9%, 6.7%)** |
| **Toddlers** |  |  |  |  |  |  |  |  |  |  |
| 1986-1990 |  |  |  |  |  |  |  |  |  |  |
| Ding *et al.* 1989 (26) | 1986 | 30486 | 28927 | 59413 | 117 | 132 | 249 | 0.4% (0.3%, 0.5%) | 0.5% (0.4%, 0.5%) | 0.4% (0.4%, 0.5%) |
| Sub-total |  | 30486 | 28927 | 59413 | 117 | 132 | 249 | 0.4% (0.3%, 0.5%) | 0.5% (0.4%, 0.5%) | 0.4% (0.4%, 0.5%) |
| 1996-2000 |  |  |  |  |  |  |  |  |  |  |
| Ding *et al.* 1998 (27) | 1996 | 43680 | 38109 | 81789 | 607 | 556 | 1163 | 1.4% (1.3%, 1.5%) | 1.5% (1.3%, 1.6%) | 1.4% (1.3%, 1.5%) |
| Sub-total |  | 43680 | 38109 | 81789 | 607 | 556 | 1163 | 1.4% (1.3%, 1.5%) | 1.5% (1.3%, 1.6%) | 1.4% (1.3%, 1.5%) |
| 2001-2005 |  |  |  |  |  |  |  |  |  |  |
| Wei *et al.* 2007 (48) | 2001 | 11024 | 10442 | 21466 | 243 | 210 | 453 | 2.2% (1.9%, 2.5%) | 2.0% (1.7%, 2.3%) | 2.1% (1.9%, 2.3%) |
| Sub-total |  | 11024 | 10442 | 21466 | 243 | 210 | 453 | 2.2% (1.9%, 2.5%) | 2.0% (1.7%, 2.3%) | 2.1% (1.9%, 2.3%) |
| 2006-2010 |  |  |  |  |  |  |  |  |  |  |
| Wang *et al.* 2008(51) | 2006 | 1844 | 1741 | 3585 | 32 | 36 | 68 | 1.7% (1.1%, 2.3%) | 2.1% (1.4%, 2.7%) | 1.9% (1.5%, 2.3%) |
| Wang *et al.* 2011(53) | 2006 | 2449 | 2383 | 4832 | 120 | 70 | 190 | 4.9% (4.0%, 5.8%) | 2.9% (2.3%, 3.6%) | 3.9% (3.4%, 4.5%) |
| NTFCOC. 2008 (28) | 2006 | 16698 | 15309 | 32007 | 1355 | 787 | 2142 | 8.1% (7.7%, 8.5%) | 5.1% (4.8%, 5.5%) | 6.7% (6.4%, 7.0%) |
| Ma *et al.* 2011 (60) | 2008-2009 | 997 | 970 | 1967 | 116 | 112 | 228 | 11.6% (9.6%, 13.6%) | 11.5% (9.5%, 13.6%) | 11.6% (10.2%, 13.0%) |
| Sub-total |  | 21988 | 20403 | 42391 | 1623 | 1005 | 2628 | 6.5% (2.8%, 10.3%) | 5.2% (3.0%, 7.4%) | 6.0% (3.0%, 8.9%) |
| **Overall** |  | **107178** | **97881** | **205059** | **2590** | **1903** | **4493** | **4.1% (2.7%, 5.4%)** | **3.2% (2.2%, 4.3%)** | **3.8% (2.6%, 5.0%)** |
| **Pre-school children** |  |  |  |  |  |  |  |  |  |  |
| 1986-1990 |  |  |  |  |  |  |  |  |  |  |
| Ding *et al.* 1989 (26) | 1986 | 26385 | 24626 | 51011 | 190 | 171 | 361 | 0.7% (0.6%, 0.8%) | 0.7% (0.6%, 0.8%) | 0.7% (0.6%, 0.8%) |
| Sub-total |  | 26385 | 24626 | 51011 | 190 | 171 | 361 | 0.7% (0.6%, 0.8%) | 0.7% (0.6%, 0.8%) | 0.7% (0.6%, 0.8%) |
| 1996-2000 |  |  |  |  |  |  |  |  |  |  |
| Ding *et al.* 1998 (27) | 1996 | 53684 | 47070 | 100754 | 1136 | 752 | 1888 | 2.1% (2.0%, 2.2%) | 1.6% (1.5%, 1.7%) | 1.9% (1.8%, 2.0%) |
| Chen *et al.* 2002(44) | 2000 | 1746 | 1611 | 3357 | 139 | 98 | 237 | 8.0% (6.7%, 9.2%) | 6.1% (4.9%, 7.3%) | 7.1% (6.2%, 7.9%) |
| Sub-total |  | 55430 | 48681 | 104111 | 1275 | 850 | 2125 | 5.0% (-0.7%, 10.7%) | 3.8% (-0.6%, 8.2%) | 4.4% (-0.6%, 9.5%) |
| 2001-2005 |  |  |  |  |  |  |  |  |  |  |
| Wei *et al.* 2007 (48) | 2001 | 15282 | 14020 | 29302 | 1186 | 830 | 2016 | 7.8% (7.3%, 8.2%) | 5.9% (5.5%, 6.3%) | 6.9% (6.6%, 7.2%) |
| Wang *et al.* 2005(46) | 2002 | 937 | 794 | 1731 | 70 | 52 | 122 | 7.5% (5.8%, 9.2%) | 6.5% (4.8%, 8.3%) | 7.0% (5.8%, 8.3%) |
| Xiang *et al.* 2005 (49) | 2004 | 2794 | 2549 | 5343 | 127 | 82 | 209 | 4.5% (3.8%, 5.3%) | 3.2% (2.5%, 3.9%) | 3.9% (3.4%, 4.4%) |
| Shan *et al.* 2010(50) | 2004 | 841 | 840 | 1681 | 33 | 26 | 59 | 3.9% (2.6%, 5.2%) | 3.1% (1.9%, 4.3%) | 3.5% (2.6%, 4.4%) |
| Sub-total |  | 19854 | 18203 | 38057 | 1416 | 990 | 2406 | 5.9% (3.8%, 8.1%) | 4.7% (2.8%, 6.5%) | 5.3% (3.4%, 7.3%) |
| 2006-2010 |  |  |  |  |  |  |  |  |  |  |
| Wang *et al.* 2008(51) | 2006 | 2141 | 2011 | 4152 | 186 | 114 | 300 | 8.7% (7.5%, 9.9%) | 5.7% (4.7%, 6.7%) | 7.2% (6.4%, 8.0%) |
| Wang *et al.* 2011(53) | 2006 | 1646 | 1650 | 3296 | 154 | 89 | 243 | 9.4% (7.9%, 10.8%) | 5.4% (4.3%, 6.5%) | 7.4% (6.5%, 8.3%) |
| NTFCOC. 2008 (28) | 2006 | 16986 | 14626 | 31612 | 1710 | 892 | 2602 | 10.1% (9.6%, 10.5%) | 6.1% (5.7%, 6.5%) | 8.2% (7.9%, 8.5%) |
| Ma *et al.* 2011 (60) | 2008-2009 | 3501 | 3185 | 6686 | 162 | 136 | 298 | 4.6% (3.9%, 5.3%) | 4.3% (3.6%, 5.0%) | 4.5% (4.0%, 5.0%) |
| Sub-total |  | 24274 | 21472 | 45746 | 2212 | 1231 | 3443 | 8.2% (5.2%, 11.2%) | 5.4% (4.4%, 6.3%) | 6.8% (4.8%, 8.8%) |
| **Overall** |  | **125943** | **112982** | **238925** | **5093** | **3242** | **8335** | **6.1% (4.4%, 7.7%)** | **4.4% (3.2%, 5.5%)** | **5.3% (3.9%, 6.7%)** |
| **School children** |  |  |  |  |  |  |  |  |  |  |
| 1981-1985 |  |  |  |  |  |  |  |  |  |  |
| Chen.1986 (30) | 1982 | 1985 | 1978 | 3963 | 6 | 4 | 10 | 0.3% (0.1%, 0.5%) | 0.2% (0.0%, 0.4%) | 0.3% (0.1%, 0.4%) |
| CNSSCH 1987 (33) | 1985 | 102696 | 102697 | 205393 | 772 | 697 | 1469 | 0.8% (0.7%, 0.8%) | 0.7% (0.6%, 0.7%) | 0.7% (0.7%, 0.8%) |
| Sub-total |  | 104681 | 104675 | 209356 | 778 | 701 | 1479 | 0.5% (0.1%, 1.0%) | 0.5% (-0.0%, 0.9%) | 0.5% (0.0%, 0.9%) |
| 1991-1995 |  |  |  |  |  |  |  |  |  |  |
| CHNS 1991 (29) | 1991 | 771 | 696 | 1467 | 14 | 16 | 30 | 1.8% (0.9%, 2.8%) | 2.3% (1.2%, 3.4%) | 2.0% (1.3%, 2.8%) |
| CNSSCH 1993(34) | 1991 | 35275 | 35030 | 70305 | 972 | 678 | 1650 | 2.8% (2.6%, 2.9%) | 1.9% (1.8%, 2.1%) | 2.3% (2.2%, 2.5%) |
| CHNS 1993 (29) | 1993 | 748 | 657 | 1405 | 19 | 19 | 38 | 2.5% (1.4%, 3.7%) | 2.9% (1.6%, 4.2%) | 2.7% (1.9%, 3.6%) |
| CNSSCH 1997(35) | 1995 | 52371 | 52540 | 104911 | 2194 | 1152 | 3346 | 4.2% (4.0%, 4.4%) | 2.2% (2.1%, 2.3%) | 3.2% (3.1%, 3.3%) |
| Sub-total |  | 89165 | 88923 | 178088 | 3199 | 1865 | 5064 | 2.9% (1.8%, 3.9%) | 2.1% (1.9%, 2.3%) | 2.6% (2.0%, 3.2%) |
| 1996-2000 |  |  |  |  |  |  |  |  |  |  |
| CHNS 1997 (29) | 1997 | 793 | 684 | 1477 | 21 | 10 | 31 | 2.6% (1.5%, 3.8%) | 1.5% (0.6%, 2.4%) | 2.1% (1.4%, 2.8%) |
| CHNS 2000 (29) | 2000 | 674 | 586 | 1260 | 21 | 8 | 29 | 3.1% (1.8%, 4.4%) | 1.4% (0.4%, 2.3%) | 2.3% (1.5%, 3.1%) |
| CNSSCH 2002(36) | 2000 | 56106 | 55990 | 112096 | 3864 | 2113 | 5977 | 6.9% (6.7%, 7.1%) | 3.8% (3.6%, 3.9%) | 5.3% (5.2%, 5.5%) |
| Zuo *et al.* 2000(42) | 1997 | 381 | 386 | 767 | 10 | 3 | 13 | 2.6% (1.0%, 4.2%) | 0.8% (-0.1%, 1.7%) | 1.7% (0.8%, 2.6%) |
| Hui *et al.* 2003(43) | 1999 | 2428 | 2146 | 4574 | 111 | 33 | 144 | 4.6% (3.7%, 5.4%) | 1.5% (1.0%, 2.1%) | 3.1% (2.6%, 3.7%) |
| Sub-total |  | 60382 | 59792 | 120174 | 4027 | 2167 | 6194 | 4.0% (1.9%, 6.1%) | 1.8% (0.3%, 3.3%) | 2.9% (1.2%, 4.7%) |
| 2001-2005 |  |  |  |  |  |  |  |  |  |  |
| Xiang *et al.* 2005 (49) | 2004 | 4588 | 4063 | 8651 | 245 | 99 | 344 | 5.3% (4.7%, 6.0%) | 2.4% (2.0%, 2.9%) | 4.0% (3.6%, 4.4%) |
| Shan *et al.* 2010(50) | 2004 | 5533 | 5254 | 10787 | 532 | 202 | 734 | 9.6% (8.8%, 10.4%) | 3.8% (3.3%, 4.4%) | 6.8% (6.3%, 7.3%) |
| CHNS 2004 (29) | 2004 | 376 | 361 | 737 | 14 | 15 | 29 | 3.7% (1.8%, 5.6%) | 4.2% (2.1%, 6.2%) | 3.9% (2.5%, 5.3%) |
| CNSSCH 2007(37) | 2005 | 58667 | 57848 | 116515 | 5819 | 2970 | 8789 | 9.9% (9.7%, 10.2%) | 5.1% (5.0%, 5.3%) | 7.5% (7.4%, 7.7%) |
| Sub-total |  | 69164 | 67526 | 136690 | 6610 | 3286 | 9896 | 7.2% (4.6%, 9.9%) | 3.9% (2.4%, 5.4%) | 5.6% (3.7%, 7.6%) |
| 2006-2010 |  |  |  |  |  |  |  |  |  |  |
| CHNS 2006 (29) | 2006 | 371 | 319 | 690 | 25 | 19 | 44 | 6.7% (4.2%, 9.3%) | 6.0% (3.4%, 8.6%) | 6.4% (4.6%, 8.2%) |
| Wang *et al.* 2008(51) | 2006 | 1587 | 1479 | 3066 | 315 | 129 | 444 | 19.8% (17.9%, 21.8%) | 8.7% (7.3%, 10.2%) | 14.5% (13.2%, 15.7%) |
| Lv *et al.* 2009 (52) | 2006 | 3123 | 2893 | 6016 | 284 | 103 | 387 | 9.1% (8.1%, 10.1%) | 3.6% (2.9%, 4.2%) | 6.4% (5.8%, 7.1%) |
| Wu *et al.* 2008(54) | 2007 | 2209 | 1931 | 4140 | 207 | 99 | 306 | 9.4% (8.2%, 10.6%) | 5.1% (4.1%, 6.1%) | 7.4% (6.6%, 8.2%) |
| Chang *et al.* 2012 (55) | 2009 | 3664 | 2860 | 6524 | 210 | 144 | 354 | 5.7% (5.0%, 6.5%) | 5.0% (4.2%, 5.8%) | 5.4% (4.9%, 6.0%) |
| Wang *et al.*2012(38) | 2010 | 300 | 300 | 600 | 53 | 41 | 94 | 17.7% (13.4%, 22.0%) | 13.7% (9.8%, 17.6%) | 15.7% (12.8%, 18.6%) |
| Liu *et al.*2012 (39) | 2010 | 600 | 600 | 1200 | 77 | 24 | 101 | 12.8% (10.2%, 15.5%) | 4.0% (2.4%, 5.6%) | 8.4% (6.8%, 10.0%) |
| Sub-total |  | 11854 | 10382 | 22236 | 1171 | 559 | 1730 | 11.4% (8.1%, 14.7%) | 6.0% (4.5%, 7.5%) | 9.0% (6.8%, 11.2%) |
| **Overall** |  | **335246** | **331298** | **666544** | **15785** | **8578** | **24363** | **6.2% (4.8%, 7.7%)** | **3.3% (2.6%, 4.1%)** | **5.0% (3.9%, 6.1%)** |
| **Adolescents** |  |  |  |  |  |  |  |  |  |  |
| 1981-1985 |  |  |  |  |  |  |  |  |  |  |
| Chen.1986 (30) | 1982 | 3349 | 2815 | 6164 | 3 | 0 | 3 | 0.1% (-0.0%, 0.2%) | 0.0% (0.0%, 0.0%) | 0.0% (-0.0%, 0.1%) |
| CNSSCH 1987 (33) | 1985 | 102404 | 102149 | 204553 | 464 | 489 | 953 | 0.5% (0.4%, 0.5%) | 0.5% (0.4%, 0.5%) | 0.5% (0.4%, 0.5%) |
| Sub-total |  | 105753 | 104964 | 210717 | 467 | 489 | 956 | 0.3% (-0.1%, 0.6%) | 0.5% (0.4%, 0.5%) | 0.3% (-0.2%, 0.7%) |
| 1991-1995 |  |  |  |  |  |  |  |  |  |  |
| CHNS 1991 (29) | 1991 | 562 | 552 | 1114 | 3 | 2 | 5 | 0.5% (-0.1%, 1.1%) | 0.4% (-0.1%, 0.9%) | 0.4% (0.1%, 0.8%) |
| CNSSCH 1993 (34) | 1991 | 35333 | 35017 | 70350 | 703 | 419 | 1122 | 2.0% (1.8%, 2.1%) | 1.2% (1.1%, 1.3%) | 1.6% (1.5%, 1.7%) |
| CHNS 1993 (29) | 1993 | 500 | 487 | 987 | 1 | 2 | 3 | 0.2% (-0.2%, 0.6%) | 0.4% (-0.2%, 1.0%) | 0.3% (-0.0%, 0.6%) |
| CNSSCH 1997 (35) | 1995 | 51589 | 52136 | 103725 | 1725 | 1046 | 2771 | 3.3% (3.2%, 3.5%) | 2.0% (1.9%, 2.1%) | 2.7% (2.6%, 2.8%) |
| Sub-total |  | 87984 | 88192 | 176176 | 2432 | 1469 | 3901 | 1.5% (0.3%, 2.7%) | 1.0% (0.4%, 1.7%) | 1.3% (0.4%, 2.2%) |
| 1996-2000 |  |  |  |  |  |  |  |  |  |  |
| CHNS 1997 (29) | 1997 | 476 | 436 | 912 | 5 | 2 | 7 | 1.1% (0.1%, 2.0%) | 0.5% (-0.2%, 1.1%) | 0.8% (0.2%, 1.3%) |
| CHNS 2000 (29) | 2000 | 542 | 488 | 1030 | 2 | 6 | 8 | 0.4% (-0.1%, 0.9%) | 1.2% (0.3%, 2.2%) | 0.8% (0.2%, 1.3%) |
| CNSSCH 2002 (36) | 2000 | 55747 | 55929 | 111676 | 3484 | 1729 | 5213 | 6.2% (6.0%, 6.5%) | 3.1% (2.9%, 3.2%) | 4.7% (4.5%, 4.8%) |
| Zuo *et al.* 2000 (42) | 1997 | 1727 | 1514 | 3241 | 182 | 77 | 259 | 10.5% (9.1%, 12.0%) | 5.1% (4.0%, 6.2%) | 8.0% (7.1%, 8.9%) |
| Sub-total |  | 58492 | 58367 | 116859 | 3673 | 1814 | 5487 | 4.5% (0.6%, 8.4%) | 2.4% (0.8%, 4.1%) | 3.5% (0.9%, 6.2%) |
| 2001-2005 |  |  |  |  |  |  |  |  |  |  |
| Xiang *et al.* 2005 (49) | 2004 | 4657 | 4641 | 9298 | 229 | 82 | 311 | 4.9% (4.3%, 5.5%) | 1.8% (1.4%, 2.1%) | 3.3% (3.0%, 3.7%) |
| Shan *et al.* 2010(50) | 2004 | 4228 | 4502 | 8730 | 281 | 114 | 395 | 6.6% (5.9%, 7.4%) | 2.5% (2.1%, 3.0%) | 4.5% (4.1%, 5.0%) |
| CHNS 2004 (29) | 2004 | 394 | 332 | 726 | 9 | 8 | 17 | 2.3% (0.8%, 3.8%) | 2.4% (0.8%, 4.1%) | 2.3% (1.2%, 3.4%) |
| CNSSCH 2007 (37) | 2005 | 58903 | 58735 | 117638 | 4725 | 2159 | 6884 | 8.0% (7.8%, 8.2%) | 3.7% (2.6%, 4.8%) | 5.9% (5.7%, 6.0%) |
| Ko *et al.* 2008 (59) | 2003-2004 | 973 | 1104 | 2077 | 54 | 41 | 95 | 5.5% (4.1%, 7.0%) | 3.7% (2.6%, 4.8%) | 4.6% (3.7%, 5.5%) |
| Sub-total |  | 69155 | 69314 | 138469 | 5298 | 2404 | 7702 | 5.6% (3.7%, 7.4%) | 2.8% (1.8%, 3.8%) | 4.2% (2.8%, 5.5%) |
| 2006-2010 |  |  |  |  |  |  |  |  |  |  |
| CHNS 2006 (29) | 2006 | 255 | 229 | 484 | 8 | 6 | 14 | 3.1% (1.0%, 5.3%) | 2.6% (0.6%, 4.7%) | 2.9% (1.4%, 4.4%) |
| Wang *et al.* 2008(51) | 2006 | 1582 | 1643 | 3225 | 233 | 192 | 425 | 14.7% (13.0%, 16.5%) | 11.7% (10.1%, 13.2%) | 13.2% (12.0%, 14.3%) |
| Chang *et al.* 2012 (55) | 2009 | 3692 | 3778 | 7470 | 152 | 148 | 300 | 4.1% (3.5%, 4.8%) | 3.9% (3.3%, 4.5%) | 4.0% (3.6%, 4.5%) |
| Cao *et al.* 2012 (56) | 2009 | 44211 | 44763 | 88974 | 3091 | 1282 | 4373 | 7.0% (6.8%, 7.2%) | 2.9% (2.7%, 3.0%) | 4.9% (4.8%, 5.1%) |
| Andegiorgish *et al.*2012(57) | 2010 | 1559 | 1581 | 3140 | 306 | 187 | 493 | 19.6% (17.7%, 21.6%) | 11.8% (10.2%, 13.4%) | 15.7% (14.4%, 17.0%) |
| Wang *et al.*2012(38) | 2010 | 300 | 300 | 600 | 33 | 23 | 56 | 11.0% (7.5%, 14.5%) | 7.7% (4.7%, 10.7%) | 9.3% (7.0%, 11.7%) |
| Liu *et al.*2012 (39) | 2010 | 600 | 600 | 1200 | 54 | 16 | 70 | 9.0% (6.7%, 11.3%) | 2.7% (1.4%, 4.0%) | 5.8% (4.5%, 7.2%) |
| Sub-total |  | 52199 | 52894 | 105093 | 3877 | 1854 | 5731 | 9.7% (6.9%, 12.6%) | 6.1% (3.9%, 8.3%) | 7.9% (5.6%, 10.3%) |
| **Overall** |  | **373583** | **373731** | **747314** | **15747** | **8030** | **23777** | **5.3% (4.1%, 6.4%)** | **3.1% (2.5%, 3.8%)** | **4.2% (3.4%, 5.1%)** |
