# Supplementary material for: Trends in Overweight and Obesity among Children and Adolescents in China from 1981 to 2010: A Meta-Analysis
Source: PLoS One. 2012 Dec 17;7(12):e51949. doi: 10.1371/journal.pone.0051949 (PMC3524084; doi:10.1371/journal.pone.0051949)
Supplement: Table S6 — Summary of studies reporting the prevalence of overweight/obesity in boys and girls in urban and rural areas. (DOC) [file pone.0051949.s009.doc]

**Table S6** Summary of studies of overweight/obesity in urban-rural and boys-girls

| Author, year | Time period  (years) | Sample size (n) | | | | Overweight (n) | | | | Obesity (n) | | | |
| --- | --- | --- | --- | --- | --- | --- | --- | --- | --- | --- | --- | --- | --- |
|  |  | Urban boys | Urban girls | Rural boys | Rural girls | Urban boys | Urban girls | Rural boys | Rural girls | Urban boys | Urban girls | Rural boys | Rural girls |
| **1991-1995** |  |  |  |  |  |  |  |  |  |  |  |  |  |
| CNSSCH 1993 (34) | 1991 | 35391 | 35147 | 35217 | 34900 | 1864 | 1408 | 934 | 1120 | 1347 | 786 | 328 | 311 |
| CHNS 1991 (29) | 1991 | 344 | 317 | 989 | 931 | 21 | 15 | 26 | 35 | 6 | 4 | 11 | 14 |
| CHNS 1993 (29) | 1993 | 312 | 288 | 936 | 856 | 21 | 15 | 43 | 33 | 4 | 9 | 16 | 12 |
| CNSSCH 1997 (35) | 1995 | 51829 | 52766 | 52131 | 51910 | 3055 | 3112 | 2026 | 2211 | 3089 | 1564 | 830 | 634 |
| **Sub-total** |  | 87876 | 88518 | 89273 | 88597 | 4961 | 4550 | 3029 | 3400 | 4446 | 2363 | 1185 | 971 |
| **1996-2000** |  |  |  |  |  |  |  |  |  |  |  |  |  |
| CHNS 1997 (29) | 1997 | 371 | 336 | 898 | 784 | 23 | 20 | 44 | 36 | 6 | 3 | 20 | 8 |
| CHNS 2000 (29) | 2000 | 327 | 313 | 889 | 761 | 31 | 15 | 55 | 36 | 8 | 5 | 15 | 9 |
| CNSSCH 2002 (36) | 2000 | 56229 | 56219 | 55624 | 55700 | 6846 | 4938 | 3690 | 3149 | 5405 | 2593 | 1943 | 1259 |
| **Sub-total** |  | 56927 | 56868 | 57411 | 57245 | 6900 | 4973 | 3789 | 3221 | 5419 | 2601 | 1978 | 1277 |
| **2001-2005** |  |  |  |  |  |  |  |  |  |  |  |  |  |
| Li *et al.* 2005 (32) | 2002 | 15856 | 15215 | 20714 | 18042 | 1238 | 816 | 697 | 569 | 644 | 424 | 353 | 263 |
| CHNS 2004 (29) | 2004 | 215 | 212 | 555 | 481 | 24 | 20 | 41 | 30 | 9 | 9 | 14 | 14 |
| CNSSCH 2007 (37) | 2005 | 59167 | 58721 | 58403 | 57862 | 8101 | 5675 | 4880 | 4130 | 7351 | 3412 | 3193 | 1717 |
| **Sub-total** |  | 75238 | 74148 | 79672 | 76385 | 9370 | 6515 | 5618 | 4725 | 8004 | 3834 | 3560 | 1994 |
| **2006-2010** |  |  |  |  |  |  |  |  |  |  |  |  |  |
| CHNS 2006 (29) | 2006 | 176 | 175 | 450 | 373 | 22 | 13 | 41 | 22 | 13 | 10 | 20 | 15 |
| Liu *et al.*2012 (39) | 2010 | 600 | 600 | 600 | 600 | 113 | 58 | 66 | 27 | 84 | 28 | 47 | 12 |
| **Sub-total** |  | 776 | 775 | 1050 | 973 | 135 | 70 | 107 | 49 | 97 | 38 | 67 | 27 |
| **Overall** |  | 440858 | 439843 | 453762 | 445427 | 42597 | 32146 | 24979 | 22741 | 35835 | 17635 | 13511 | 8510 |
