# Supplementary material for: Trends in Overweight and Obesity among Children and Adolescents in China from 1981 to 2010: A Meta-Analysis
Source: PLoS One. 2012 Dec 17;7(12):e51949. doi: 10.1371/journal.pone.0051949 (PMC3524084; doi:10.1371/journal.pone.0051949)
Supplement: Appendix S1 — Search strategy for CNKI, Wanfang DATA, CINAHL, EMBASE and MEDLINE databases. (DOC) [file pone.0051949.s010.doc]

**Appendix S1**

**Search strategy for CNKI, Wanfang DATA, CINAHL, EMBASE and MEDLINE databases**

*Search strategy for CNKI and Wanfang DATA databases*

China National Knowledge Infrastructure (CNKI; http://www.cnki.net) is the largest Chinese database, covering more than 9000 Chinese journals on different subjects such as public health, medicine and social science, with 50 million full-text articles in Chinese. The earliest papers included in the database are from 1979. Wanfang DATA (http://www.wanfangdata.com.cn) contains full-text articles from more than 5500 core Chinese language journals on different subjects. The journals published by the Chinese Medical Association are exclusively included in Wanfang DATA since 2008. The same search strategy was used for both the CNKI and Wanfang DATA databases.

Search strategy:

1. overweight .kw.

2. obesity .kw.

3. body mass index .kw.

4. BMI .kw.

5. weight gain .kw.

6. or/1-5

7. incidence .kw.

8. frequency .kw.

9. prevalence .kw.

10. epidemiology .kw.

11. or/7-10

12. China .kw.

13. Chinese .kw.

14. or/12-13

15. infant .kw.

16. childhood .kw.

17. children .kw.

18. toddler .kw.

19. adolescence .kw.

20. youth .kw.

21. teen .kw.

22. teenager .kw.

23. or/15-22

24. 6 and 11 and 14 and 23

*Search strategy for CINAHL, EMBASE and MEDLINE*

The CINAHL database was searched using the OVID interface (http://www.ovid.com). EMBASE and MEDLINE were searched using the EMBASE interface (http://www.embase.com). The same search strategy was used for all three databases.

Search strategy:

1. exp OBESITY/

2. exp Weight Gain/

3. obes$.af.

4. weight gain.af.

5. (overweight or over weight).af.

6. (bmi or body mass index).af.

7. or/1-6

8. exp incidence/

9. exp frequency/

10. exp prevalence/

11. exp epidemiology/

12. or/8-11

13. exp China/

14. exp Chinese/

15. or/13-14

16. infant$.af.

17. child$.af.

18. teen$.af.

19. exp toddler/

20. exp adolescence/

21. exp youth

22. or/13-21

23. 7 and 12 and 15 and 22

24. Animals/

25. 23 not 24

26. limit 25 to yr=1970-2012
