# Supplementary material for: Trends in Overweight and Obesity among Children and Adolescents in China from 1981 to 2010: A Meta-Analysis
Source: PLoS One. 2012 Dec 17;7(12):e51949. doi: 10.1371/journal.pone.0051949 (PMC3524084; doi:10.1371/journal.pone.0051949)
Supplement: Appendix S2 — Quality-assessment extraction form. (DOC) [file pone.0051949.s011.doc]

**Appendix S2**

Quality-assessment extraction form

| Assessment items | Score |
| --- | --- |
| **1. Study design** |  |
| Prospective  Retrospective  No information | 2  1  0 |
| **2. Representativeness of target population** |  |
| Entire target population with nationally representative samples | 2 |
| An entire state or province  A specified number of sampling units (i.e., cities or towns, clinics)  **3. Sample selection**  Multistage, stratified, random sampling  Two- or one-stage, stratified, random sampling  Non-random sampling  **4. Sample size**  >10,000  5,000–10,000  <5,000 | 1  0  2  1  0  2  1  0 |
| **5. Response rate** |  |
| >90% | 2 |
| 70–90% | 1 |
| <70% | 0 |
| **6. Reasons for nonresponse** |  |
| Detailed description | 2 |
| Unclear description | 1 |
| No information | 0 |
| **7. Data source and study objectives** |  |
| Primarily from a national study on prevalence of overweight/obesity | 2 |
| Primarily from a state or provincial study on prevalence of overweight/obesity | 1 |
| Primarily from a study on prevalence of overweight/obesity in small sampling units (i.e., cities or towns, clinics) or a survey not specifically designed for the study of the prevalence of overweight/obesity | 0 |
| **8. Data collection** |  |
| Timely measurement and recording | 2 |
| A validated questionnaire/interview  Retrospectively from records  **9. Description of obesity, overweight, sex, age, urban and rural**  All points  3–4 points  1–2 points  **10. Prevalence recall periods**  Stated  No information | 1  0  2  1  0  1  0 |

Interpretation: score >14 (maximum, 19), high quality; 11–14, medium quality; <11, low quality
