# Supplementary material for: Trends in Overweight and Obesity among Children and Adolescents in China from 1981 to 2010: A Meta-Analysis
Source: PLoS One. 2012 Dec 17;7(12):e51949. doi: 10.1371/journal.pone.0051949 (PMC3524084; doi:10.1371/journal.pone.0051949)
Supplement: Appendix S3 — General information on and study designs of the four included national surveys. (DOC) [file pone.0051949.s012.doc]

**Appendix S3**

**General information on and study designs of the four included national surveys**

Four national surveys have successively described the prevalence of overweight/obesity; their background and study designs have been described below.

**National Survey on Childhood Obesity (NSCO)**

From 1986, the National Survey on Childhood Obesity (NSCO) has been conducted in China to understand the status and trends of overweight/obesity among children aged 0–7 years, by the National Task Force on Childhood Obesity of China (NTFCOC). Thus far, the survey has been carried out 3 times (1986, 1996 and 2006). A total of 431,308 children aged 0–7 years have been recruited in the three surveys, by means of random cluster sampling from 8–11 cities in the northern, central, southern and western regions of China.

In 2006, the prevalence of obesity was 7.2% in all subjects, 8.9% in boys and 5.3% in girls; these values are 9.0, 11.1 and 7.6 times higher respectively than those in 1986. They represent an average annual increase rate of 32%, 41% and 23%, respectively.

In the first NSCO in 1986, the prevalence of overweight was not reported. In 2006, the prevalence of overweight was 19.8% in all subjects, 22.2% in boys and 17.0% in girls; these values are 4.7, 5.3 and 4.1 times higher respectively than those in 1996. They represent an average annual increase rate of 156%, 180% and 129%, respectively.

**China Health and Nutrition Survey (CHNS)**

The China Health and Nutrition Survey (CHNS) were designed to monitor the health and nutritional status of the Chinese population. This survey has been conducted successively in 1989, 1991, 1993, 1997, 2000, 2004, 2006 and 2009. The study sample was drawn from nine provinces (Liaoning, Heilongjiang, Jiangsu, Shandong, Henan, Hubei, Hunan, Guangxi and Guizhou) by means of a multistage, random cluster process. The study was approved by the institutional review board of the University of North Carolina at Chapel Hill and the National Institute for Nutrition and Food Safety, Chinese Centers for Disease Control and Prevention. CHNS data can be obtained from the website <http://www.cpc.unc.edu/projects/china/data/data.html>.

In the 1989 CHNS, the prevalence of overweight/obesity in children and adolescents aged 0–18 years was not reported. Furthermore, data for 2009 could not been obtained from the CHNS website. We therefore included data from six surveys (1991, 1993, 1997, 2000, 2004 and 2006) in our review. A total of 12,289 children and adolescents aged 7–18 years were recruited in the six surveys.

In 2006, the prevalence of overweight was 8.3% in all subjects, 10.1% in boys and 6.4% in girls; these values are 2.2, 2.9 and 1.6 times higher respectively than those in 1991. They represent an average annual increase rate of 28%, 41% and 15%, respectively. In 2006, the prevalence of obesity was 4.9% in all subjects, 5.3% in boys and 4.6% in girls; these values are 3.5, 3.3 and 4.1 times higher respectively than those in 1991. They represent an average annual increase rate of 22%, 20% and 25%, respectively.

**Chinese National Nutrition and Health Survey (CNNHS)**

Three China National Nutrition and Health Surveys (CNNHSs) have been conducted (in 1982, 1992 and 2002) by the ethics committee of the National Institute for Nutrition and Food Safety, Chinese Center for Disease Control and Prevention. The three surveys covered 25–31 provinces or municipalities, and all administrative units directly under the control of the central government of China. A multistage, cluster sampling method was used for subject selection. In 1982, the survey reported the prevalence of overweight/obesity in children and adolescents aged 7–18 years (data for children aged 0–7 years was not reported). In 1992 and 2002, the survey reported the prevalence of overweight/obesity in children and adolescents aged 0–18 years.

A total of 33,966 children aged 0–7 years were recruited in the 1992 and 2002 surveys. In 2002, the prevalence of overweight was 3.4% in all subjects aged 0–7 years, 3.4% in boys and 3.4% in girls; these values are 1.5, 1.5 and 1.4 times higher respectively than those in 1992. They represent an average annual increase rate of 11%, 10% and 12%, respectively. In 2002, the prevalence of obesity was 2.0% in all subjects aged 0–7 years, 2.0% in boys and 2.1% in girls; these values are 1.2, 1.2 and 1.3 times higher respectively than those in 1992. They represent an average annual increase rate of 3%, 3% and 5%, respectively.

A total of 71,226 children and adolescents aged 7–18 years were recruited in the three surveys. In 2002, the prevalence of overweight was 4.3% in all subjects aged 7–18 years, 4.9% in boys and 3.7% in girls; these values are 3.9, 4.9 and 2.8 times higher respectively than those in 1982. They represent an average annual increase rate of 16%, 20% and 12%, respectively. In 2002, the prevalence of obesity was 2.1% in all subjects aged 7–18 years, 2.5% in boys and 1.7% in girls; these values are 21, 17 and 13 times higher respectively than those in 1982. They represent an annual increase rate of 10%, 13% and 8%, respectively.

**Chinese National Survey on Students Constitution and Health** **(CNSSCH)**

The Chinese National Survey on Student’s Constitution and Health (CNSSCH) has been conducted every 5 years since 1985 by the Ministry of Education, the Ministry of Health, the Ministry of Science and Technology, the State of Nation Affairs and the State Sports General Administration, People’s Republic of China. It is, thus far, the largest, nationally representative sample of school-aged children and adolescents in China. It has been widely used to produce national and state prevalence estimates for a variety of health indicators of school-aged children.

This survey has been carried out 6 times (1985, 1991, 1995, 2000, 2005 and 2010). Detailed data from the 2010 CNSSCH is not still being published and was not included in our review. A total of 1,217,162 children aged 7–18 years were recruited in the other five surveys, by means of random, two-stage, stratified cluster sampling from 30 provinces in China. In 2005, the prevalence of overweight was 9.7% in all subjects, 11.0% in boys and 8.4% in girls; these values are 3.6, 5.2 and 3.0 times higher respectively than those in 1985. They represent an average annual increase rate of 36%, 45% and 28%, respectively. In 2005, the prevalence of obesity was 6.7% in all subjects, 9.0% in boys and 4.4% in girls; these values are 11.2, 15.0 and 7.3 times higher respectively than those in 1985. They represent an average annual increase rate of 31%, 42% and 19%, respectively.
