# Supplementary material for: Trends in Overweight and Obesity among Children and Adolescents in China from 1981 to 2010: A Meta-Analysis
Source: PLoS One. 2012 Dec 17;7(12):e51949. doi: 10.1371/journal.pone.0051949 (PMC3524084; doi:10.1371/journal.pone.0051949)
Supplement: Appendix S4 — Quality assessment (grade) of the 35 included papers (41 studies). (DOC) [file pone.0051949.s013.doc]

**Appendix S4** Quality assessment (grade) of the 35 included papers (41 studies).

| **Author, year** | **Design of study** | **Target population** | **Sample selection** | **Sample size** | **Response rate** | **Reasons for nonresponse** | **Data source and study objectives** | **Data collection** | **Description information** | **Prevalence recall periods** | **Total** |
| --- | --- | --- | --- | --- | --- | --- | --- | --- | --- | --- | --- |
| **National Survey on Childhood Obesity** | | | | | | | | | | | |
| Ding *et al.* 1989 (26) | 2 | 2 | 1 | 2 | 2 | 0 | 2 | 2 | 1 | 1 | 15 |
| Ding *et al.* 1998 (27) | 2 | 2 | 1 | 2 | 2 | 0 | 2 | 2 | 1 | 1 | 15 |
| NTFCOC. 2008 (28) | 2 | 2 | 1 | 2 | 2 | 0 | 2 | 2 | 1 | 1 | 15 |
| **China Health and Nutrition Survey** | | | | | | | | | | | |
| CHNS (1991/1993/1997/  2000/2004/2006) (29) | 2 | 2 | 1 | 0 | 2 | 0 | 0 | 2 | 2 | 1 | 12 |
| **Chinese national nutrition and health survey** | | | | | | | | | | | |
| Chen.1986 (30) | 2 | 2 | 1 | 2 | 2 | 0 | 0 | 2 | 2 | 1 | 14 |
| Ge.1995 (31) | 2 | 2 | 1 | 2 | 2 | 0 | 0 | 2 | 2 | 1 | 14 |
| Li *et al.* 2005 (32) | 2 | 2 | 1 | 2 | 2 | 0 | 0 | 2 | 2 | 1 | 14 |
| **Chinese National Survey on Students Constitution and Health** | | | | | | | | | | | |
| CNSSCH 1987 (33) | 2 | 2 | 1 | 2 | 2 | 0 | 2 | 2 | 2 | 1 | 16 |
| CNSSCH 1993 (34) | 2 | 2 | 1 | 2 | 2 | 0 | 2 | 2 | 2 | 1 | 16 |
| CNSSCH 1997 (35) | 2 | 2 | 1 | 2 | 2 | 0 | 2 | 2 | 2 | 1 | 16 |
| CNSSCH 2002 (36) | 2 | 2 | 1 | 2 | 2 | 0 | 2 | 2 | 2 | 1 | 16 |
| CNSSCH 2007 (37) | 2 | 2 | 1 | 2 | 2 | 0 | 2 | 2 | 2 | 1 | 16 |
| **Sixth Chinese National Survey on Students Constitution and Health (2010)** | | | | | | | | | | | |
| Wang *et al.*2012 (38) | 2 | 1 | 1 | 0 | 2 | 0 | 1 | 2 | 1 | 1 | 11 |
| Liu *et al.*2012 (39) | 2 | 0 | 1 | 0 | 2 | 0 | 0 | 2 | 1 | 1 | 9 |
| Zhang (a) *et al.*2012 (40) | 2 | 1 | 1 | 1 | 2 | 0 | 1 | 2 | 1 | 1 | 12 |
| **Regional Survey on Childhood Obesity** | | | | | | | | | | | |
| Ding *et al.* 1988 (41) | 1 | 1 | 2 | 1 | 2 | 0 | 1 | 2 | 1 | 0 | 11 |
| Zuo *et al.* 2000 (42) | 1 | 0 | 1 | 0 | 2 | 0 | 0 | 2 | 1 | 0 | 7 |
| Hui *et al.* 2003 (43) | 1 | 1 | 1 | 0 | 2 | 0 | 0 | 2 | 1 | 0 | 8 |
| Chen *et al.* 2002 (44) | 1 | 1 | 2 | 0 | 2 | 0 | 1 | 2 | 1 | 0 | 10 |
| Li *et al.* 2007 (45) | 1 | 0 | 2 | 1 | 2 | 0 | 0 | 2 | 1 | 0 | 9 |
| Wang *et al.* 2005 (46) | 1 | 0 | 2 | 0 | 2 | 0 | 0 | 2 | 1 | 0 | 8 |
| Zhang *et al.* 2003 (47) | 1 | 1 | 2 | 1 | 2 | 0 | 1 | 2 | 2 | 0 | 12 |
| Wei *et al.* 2007 (48) | 1 | 1 | 2 | 2 | 2 | 0 | 1 | 2 | 1 | 0 | 12 |
| Xiang *et al.* 2005 (49) | 1 | 1 | 2 | 2 | 2 | 0 | 1 | 2 | 1 | 0 | 12 |
| Shan *et al.* 2010 (50) | 1 | 1 | 1 | 2 | 2 | 0 | 0 | 2 | 1 | 0 | 10 |
| Wang *et al.* 2008 (51) | 1 | 1 | 2 | 2 | 2 | 0 | 1 | 2 | 1 | 0 | 12 |
| Lv *et al.* 2009 (52) | 1 | 0 | 2 | 1 | 2 | 0 | 0 | 2 | 1 | 0 | 9 |
| Wang *et al.* 2011 (53) | 1 | 0 | 2 | 1 | 2 | 0 | 0 | 2 | 1 | 0 | 9 |
| Wu *et al.* 2008 (54) | 1 | 0 | 2 | 0 | 2 | 0 | 0 | 2 | 1 | 0 | 8 |
| Chang *et al.* 2012 (55) | 1 | 0 | 2 | 2 | 2 | 0 | 0 | 2 | 1 | 0 | 10 |
| Cao *et al.* 2012 (56) | 1 | 0 | 2 | 2 | 2 | 0 | 0 | 2 | 1 | 0 | 10 |
| Andegiorgish *et al.* 2012 (57) | 1 | 1 | 2 | 2 | 2 | 0 | 0 | 2 | 1 | 0 | 9 |
| Zhang (b) *et al.* 2012 (58) | 1 | 1 | 2 | 2 | 2 | 0 | 1 | 2 | 1 | 0 | 12 |
| Ko *et al.* 2008 (59) | 1 | 1 | 2 | 0 | 2 | 0 | 1 | 2 | 1 | 0 | 10 |
| Ma *et al.* 2011 (60) | 1 | 0 | 2 | 1 | 2 | 0 | 0 | 2 | 1 | 0 | 9 |
