# Supplementary material for: Trends in Overweight and Obesity among Children and Adolescents in China from 1981 to 2010: A Meta-Analysis
Source: PLoS One. 2012 Dec 17;7(12):e51949. doi: 10.1371/journal.pone.0051949 (PMC3524084; doi:10.1371/journal.pone.0051949)
Supplement: Appendix S5 — Sensitivity analysis of the studies on the prevalence of overweight/obesity in children and adolescents. (DOC) [file pone.0051949.s014.doc]

**Appendix S5** Sensitivity analysis of the studies on the prevalence of overweight/obesity in children and adolescents

|  |  |  | Overweight |  |  | Obesity |  |
| --- | --- | --- | --- | --- | --- | --- | --- |
| Study characteristic | Number of studies (n) | ES (95% CI) | *x^2^* | *P* | ES (95% CI) | *x^2^* | *P* |
| **Sample size** |  |  |  |  |  |  |  |
| >10000 | 5 | 14.3% (10.9%-17.7%) | 7.48 | 0.006 | 5.9% (4.7%-7.1%) | 25.87 | 0.000 |
| ≤10000 | 9 | 12.4% (10.7%-14.2%) | 82.74 | 0.000 | 8.5 % (6.7%-10.3%) | 265.57 | 0.000 |
| **Quality grade of study** |  |  |  |  |  |  |  |
| High | 1 | 19.8% (19.5%-20.1%) | 1185.70 | 0.000 | 7.2% (7.0%-7.4%) | 175.42 | 0.000 |
| Medium | 5 | 13.2% (11.8%-14.7%) | 0.87 | 0.350 | 8.5% (6.7%-10.3%) | 12.94 | 0.0003 |
| Low | 8 | 12.3% (10.9%-13.6%) | 768.04 | 0.000 | 8.2% (5.5%-11.0%) | 47.61 | 0.000 |
| **Diagnostic criteria for obesity** |  |  |  |  |  |  |  |
| BMI (WGOC) | 7 | 12.0% (10.7%-13.3%) | 803.33 | 0.000 | 8.8% (6.6%-9.8%) | 40.92 | 0.000 |
| BMI (IOTF) | 4 | 12.4% (9.7%-15.2%) | 0.16 | 0.690 | 6.8% (4.0%-9.5%) | 79.68 | 0.000 |
| BMI (CDC) | 1 | 14.2% (13.7%-14.7%) | 6.93 | 0.009 | 8.0% (7.6%-8.3%) | 121.4 | 0.000 |
| W/IW (WHO) | 2 | 17.7% (13.4%-21.9%) | 1063.58 | 0.000 | 6.4% (4.8%-8.0%) | 142.72 | 0.000 |
| **Geographical distribution** |  |  |  |  |  |  |  |
| National | 2 | 6.1% (4.0%-8.3%) | 168.70 | 0.000 | 14.1% (2.9%-25.3%) | 1123.83 | 0.000 |
| Coastal big city | 3 | 10.9% (2.7%-19.1%) | 102.30 | 0.000 | 13.6% (11.2%-15.9%) | 6.87 | 0.009 |
| Inland big city | 3 | 7.8% (4.5%-11.1%) | 115.88 | 0.000 | 15.5% (14.6%-16.4%) | 4.07 | 0.044 |
| Western city | 6 | 6.3% (5.2%-7.5%) | 39.03 | 0.000 | 11.4% (10.2%-12.5%) | 848.41 | 0.000 |
