# Supplementary material for: Trends in Overweight and Obesity among Children and Adolescents in China from 1981 to 2010: A Meta-Analysis
Source: PLoS One. 2012 Dec 17;7(12):e51949. doi: 10.1371/journal.pone.0051949 (PMC3524084; doi:10.1371/journal.pone.0051949)
Supplement: Appendix S6 — Funnel Plot and Begg test for meta-analysis of overweight in boys compared with girls (ages, 0–18 years). (DOC) [file pone.0051949.s015.doc]

**Appendix S6**

Funnel plot and Begg test for meta-analysis of overweight in boys compared with girls (ages, 0–18 years)

**
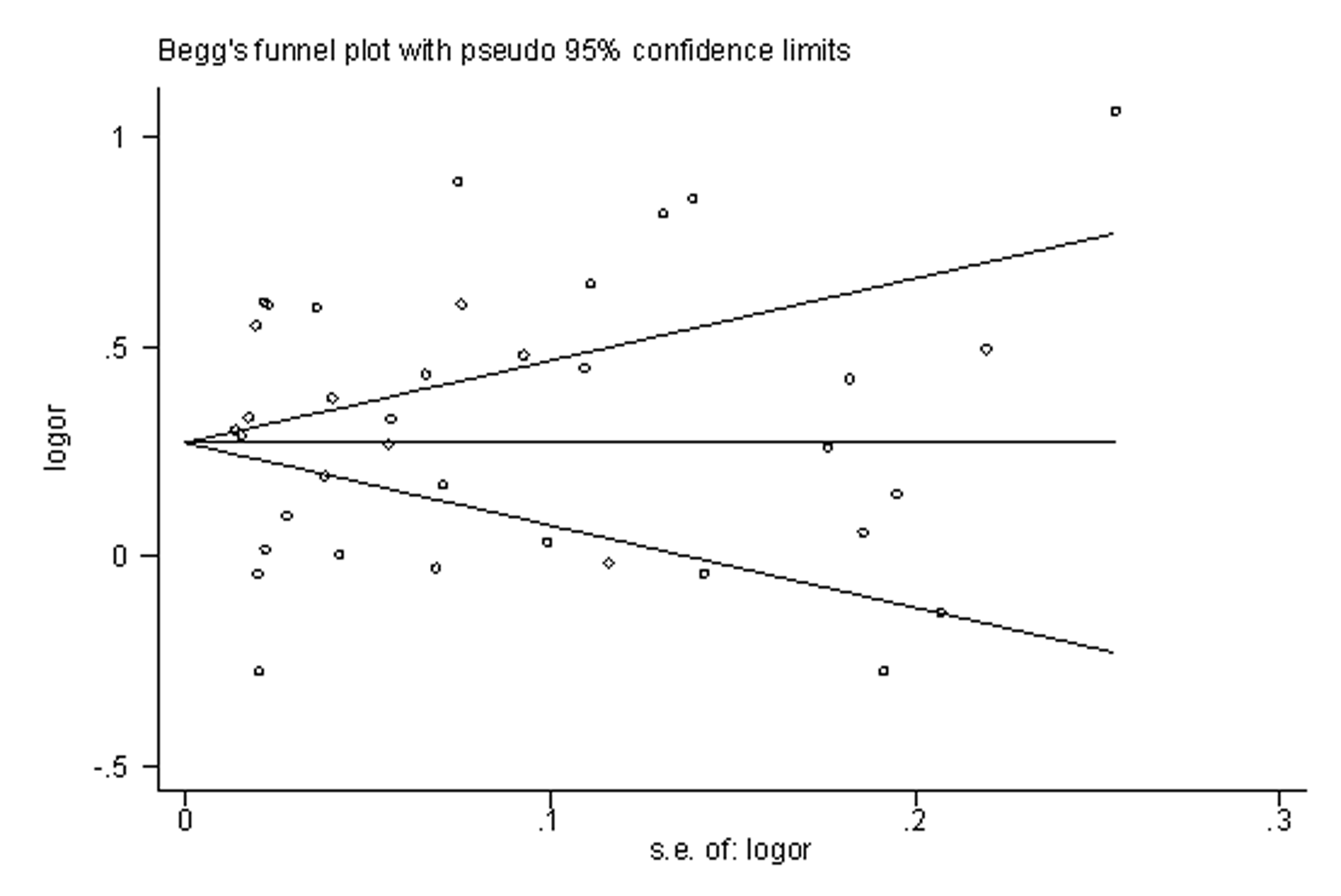
**

Tests for publication bias

Begg test

adj. Kendall score (P-Q) = -28

Standard deviation of score = 76.46

Number of studies = 37

z = -0.37

Pr > |z| = 0.714

z = 0.35 (continuity corrected)

Pr > |z| = 0.724 (continuity corrected)

Egger's test

----------------------------------------------------------------------------------------------------------------------

Std_Eff | Coef. Std. Err. t P>|t| [95% Conf. Interval]

-------------+--------------------------------------------------------------------------------------------------------

slope | .2511551 .0665708 3.77 0.001 .1160092 .3863011

bias | .7604656 1.974254 0.39 0.702 -3.247483 4.768414

----------------------------------------------------------------------------------------------------------------------
