# Supplementary material for: Trends in Overweight and Obesity among Children and Adolescents in China from 1981 to 2010: A Meta-Analysis
Source: PLoS One. 2012 Dec 17;7(12):e51949. doi: 10.1371/journal.pone.0051949 (PMC3524084; doi:10.1371/journal.pone.0051949)
Supplement: Appendix S7 — Funnel Plot and Begg test of for meta-analysis of obesity in boys compared with girls (ages, 0–18 years). (DOC) [file pone.0051949.s016.doc]

**Appendix S7**

Funnel plot and Begg test for meta-analysis of obesity in boys compared with girls (ages, 0–18 years)


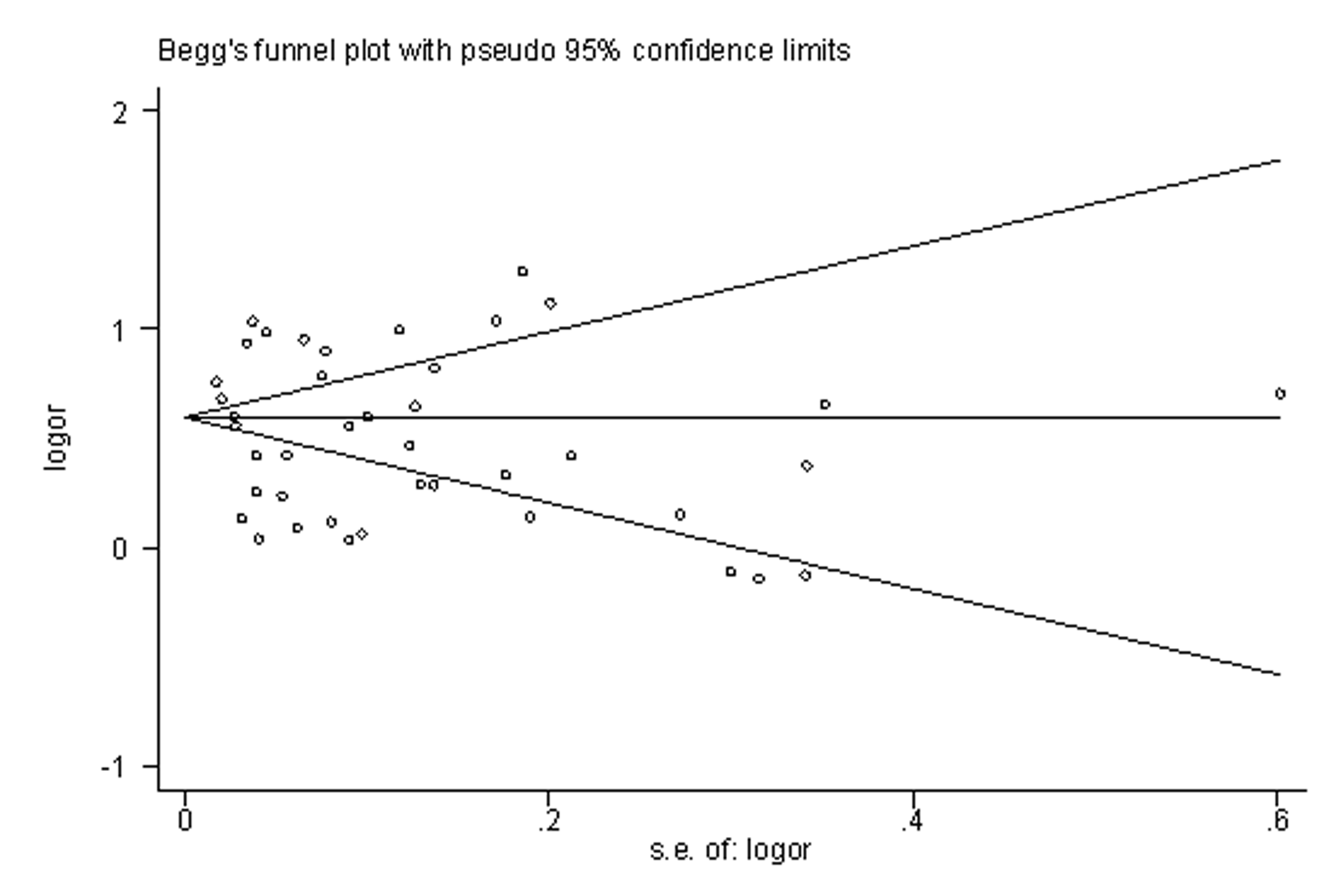


Tests for publication bias

Begg test

adj. Kendall score (P-Q) = -8

Standard deviation of score = 89.03

Number of studies = 41

z = -0.09

Pr > |z| = 0.928

z = 0.08 (continuity corrected)

Pr > |z| = 0.937 (continuity corrected)

Egger's test

----------------------------------------------------------------------------------------------------------------------

Std_Eff | Coef. Std. Err. t P>|t| [95% Conf. Interval]

-------------+--------------------------------------------------------------------------------------------------------

slope | .6585608 .0679893 9.69 0.000 .5210395 .7960821

bias | -1.590095 1.34479 -1.18 0.244 -4.310189 1.129999

----------------------------------------------------------------------------------------------------------------------
